# Synthesis, Biological Evaluation, and Molecular Docking Studies of Novel Coumarin–Triazole–Isatin Hybrids as Selective Butyrylcholinesterase Inhibitors

**DOI:** 10.3390/molecules30102121

**Published:** 2025-05-11

**Authors:** Aleksandar Dimkovski, Vladimir Dobričić, Milena R. Simić, Maja Jurhar Pavlova, Evgenija Mihajloska, Zoran Sterjev, Ana Poceva Panovska

**Affiliations:** 1 Institute for Pharmaceutical Chemistry, Faculty of Pharmacy, Ss Cyril and Methodius University in Skopje, 1000 Skopje, North Macedonia; emihajloska@ff.ukim.edu.mk (E.M.); zost@ff.ukim.edu.mk (Z.S.); 2 Department of Pharmaceutical Chemistry, Faculty of Pharmacy, University of Belgrade, 11221 Belgrade, Serbia; 3 Department of Organic Chemistry, Faculty of Pharmacy, University of Belgrade, 11221 Belgrade, Serbia; milena.simic@pharmacy.bg.ac.rs; 4 Institute for Microbiology and Parasitology, Faculty of Medicine, Ss Cyril and Methodius University in Skopje, 1000 Skopje, North Macedonia; maja.jurhar@medf.ukim.edu.mk; 5 Institute for Applied Chemistry and Pharmaceutical Analysis, Faculty of Pharmacy, Ss Cyril and Methodius University in Skopje, 1000 Skopje, North Macedonia; anpo@ff.ukim.edu.mk

**Keywords:** coumarin–trazole–isatin derivatives, Alzheimer’s disease, cholinesterase, hybrid molecules, molecular docking

## Abstract

A series of 21 novel coumarin–triazole–isatin hybrids was synthesized and evaluated for their potential as multitarget agents in Alzheimer’s disease (AD). The compounds featured variations in alkyl linker length that connects coumarin and triazole and substitution at the 5-position of the isatin ring. Several derivatives showed potent butyrylcholinesterase (BChE) inhibition with selectivity over acetylcholinesterase (AChE). The lead compound, **6c1**, exhibited strong BChE inhibition (IC_50_ = 1.74 μM), surpassing donepezil. Enzyme kinetics revealed a mixed-type mechanism, while molecular docking studies confirmed dual binding at catalytic and peripheral sites. Structure–activity relationship (SAR) analysis highlighted the influence of linker flexibility and steric/electronic effects of substituents. The observed BChE selectivity, combined with favorable in vitro profiles, identifies these hybrids as promising leads for AD drug development.

## 1. Introduction

Alzheimer’s disease (AD) is a progressive neurodegenerative disorder that leads to gradual cognitive impairment, memory loss, and synaptic dysfunction [1]. As the most common cause of dementia, it represents a major global health burden. According to the WHO 2022 Dementia Research Blueprint, dementia currently affects 55.2 million people worldwide, and this number is projected to rise to 78 million by 2030. The economic burden of the disease, including direct medical costs and indirect care expenses, is estimated to be well over 2.5 trillion USD [2]. Despite intensive research efforts, the precise etiology of AD remains unclear. However, growing evidence suggest that AD is a multifactorial disorder driven by amyloid-β aggregation, tau hyperphosphorylation, neuroinflammation, oxidative stress, and cholinergic dysfunction [3,4,5,6,7].

Of the many pathological mechanisms involved in AD, cholinergic dysfunction is one of the most recognized contributors to cognitive decline. The cholinergic system, which plays a key role in memory, learning, and synaptic plasticity, gradually deteriorates as the disease progresses. This decline is primarily due to the degeneration of cholinergic neurons in critical brain regions, leading to a substantial drop in acetylcholine (ACh) levels, which further exacerbates cognitive impairment [8,9]. As a result, therapeutic strategies for AD have largely focused on inhibiting the enzymes responsible for ACh hydrolysis to help restore cholinergic function [10].

The two main enzymes involved in ACh breakdown are acetylcholinesterase (AChE) and butyrylcholinesterase (BChE). AChE is the primary enzyme regulating cholinergic neurotransmission, rapidly hydrolyzing ACh at synaptic junctions. It is found at significant levels in the membranes of red blood cells and nervous system, particularly at neuromuscular junctions and brain areas associated with memory and cognition such as the hippocampus, basal forebrain, and neocortex [11]. BChE, in contrast, is a non-specific cholinesterase, capable of hydrolyzing a broader range of choline esters. It is widely distributed throughout the body, with its highest concentrations in blood plasma and liver, and other peripheral tissues. In the central nervous system (CNS), BChE is predominantly expressed by glial cells and white matter in the brain [12,13].

In the past, BChE was considered less important than AChE due to its lower expression in the brain. However, it is now well established that as AD progresses, cholinesterase activity changes significantly, with BChE taking on an increasingly dominant role in ACh hydrolysis. In the early stages of AD, AChE is the primary enzyme responsible for degrading nearly 80% of synaptic ACh. However, as neuronal degeneration advances, AChE activity declines significantly, decreasing by up to 85% in key brain regions such as the hippocampus and cerebral cortex. In contrast, BChE expression progressively increases, rising two- to four-fold, which alters the BChE/AChE ratio from approximately 0.2 to over 11 in some brain areas [14,15,16].

AChE and BChE, as serine esterases, exhibit structural and functional similarities but differ in their efficiency and selectivity for substrate hydrolysis. Both enzymes share three key structural regions: the catalytic active site (CAS), the gorge, and the peripheral anionic site (PAS). The CAS contains the Ser-Glu-His triad, along with essential structural components, including the anionic subsite, oxyanion hole, and acyl pocket, all of which are critical for enzymatic function. The gorge is about 20 Å deep and links the CAS in the enzyme’s interior to the PAS near the entrance of the active site, facilitating substrate movement and enzyme regulation. The PAS is essential for substrate recognition, enzyme regulation and ligand binding, influencing the access to the CAS and modulating interactions with inhibitors and amyloid-β aggregation in AD [17,18]. The key difference between AChE and BChE is in their active sites, which influences substrate specificity. In AChE, the acyl pocket is tightly restricted by two phenylalanine (Phe) residues, forming a narrow binding site that efficiently breaks down ACh but prevents larger molecules from entering. In contrast, BChE has leucine (Leu) and valine (Val) instead of Phe, creating a more open and flexible site that accommodates bulkier substrates. Additionally, AChE contains more aromatic residues in the gorge, supporting π–π interactions, whereas BChE has hydrophobic residues, allowing for greater substrate versatility [19,20].

Current AD treatment options focus on enhancing cholinergic neurotransmission and modulating glutamate signaling to improve cognition and manage symptoms. Cholinesterase inhibitors (ChEIs)—donepezil, galantamine, and rivastigmine—increase ACh levels by inhibiting its enzymatic breakdown [21,22], while *N*-methyl-D-aspartate (NMDA) receptor antagonists, such as memantine, help regulate glutamate-mediated excitotoxicity [22]. Monoclonal antibodies such as aducanumab, lecanemab, and donanemab have emerged as the first disease-modifying therapies for AD by targeting and clearing amyloid-β plaques, but their modest cognitive benefits, serious side effects, and high costs [23,24] have limited their widespread use, leaving cholinesterase inhibitors and NMDA antagonists as the mainstay of AD treatment.

Given that BChE contributes to ACh hydrolysis and has been implicated in amyloid pathology, as it is found in mature amyloid-β plaques [13] and may contribute to amyloid-β aggregation by facilitating peptide fibrillization [25], interest in dual AChE/BChE inhibitors has grown as a potential strategy for AD treatment. Additionally, selective BChE inhibitors may avoid side effects associated with AChE inhibition, such as gastrointestinal disturbances and bradycardia [26,27].

Coumarin, isatin, and several of their derivatives are classified as secondary metabolites, commonly identified and isolated from various families of plants, fungi, and bacteria. Coumarin-based compounds are typically known to exert their inhibitory effects on cholinesterases by interacting with the peripheral anionic site (PAS) of the enzyme. However, in certain cases, especially in hybrid molecules, coumarin can also engage with the catalytic active site (CAS), which is influenced by the molecular structure and additional functional groups incorporated into the hybrid design [28,29,30,31,32]. Recent studies have further emphasized the ability of isatin and its derivatives to inhibit cholinesterase activity primarily through interactions with the CAS [33,34]. This structural flexibility allows for more selective and potent inhibitors in hybrid designs, enhancing their potential for therapeutic applications. Molecules incorporating either the coumarin or isatin scaffold display a broad spectrum of pharmacological properties, including anticoagulant, antibacterial, antifungal, antitubercular, anti-HIV, antioxidant, antihypertensive, anticonvulsant, antihyperglycemic, and anticancer activities [35,36,37,38,39]. Extensive literature data support the cholinesterase inhibitory potential of both coumarin and isatin derivatives [40,41,42,43,44,45].

Triazole is an *N*-heterocyclic compound known for its broad spectrum of biological activities, including antiproliferative, anticonvulsant, antimicrobial, antineoplastic, antiviral, analgesic, and anti-inflammatory effects [46,47]. Due to its unique structure and electronic characteristics, incorporation of the triazole moiety into potential drug candidates has proven especially valuable for enhancing potency or selectivity. Additionally, triazoles can be readily synthesized via click chemistry and exhibit high stability toward oxidation, reduction, and hydrolysis, making them ideal linkers for joining two distinct pharmacophores within a single drug molecule [48]. Literature reports further highlight the triazole ring as an active pharmacophore with notable potency and selectivity toward AChE [49].

Given the potential advantages of targeting multiple subsites within cholinesterases, we developed a novel series of coumarin–triazole–isatin hybrids, aiming to enhance both cholinesterase inhibition and modulate amyloid aggregation in AD. These scaffolds integrate three pharmacophores with distinct and complementary binding characteristics, designed to interact with different regions of the enzyme active site. To explore structure–activity relationships (SAR), we systematically modified two structural features: the length of the alkyl linker bridging the coumarin and triazole rings (ranging from 2 to 4 carbon atoms) and the nature of the substituent at the C-5 position of the isatin moiety (including H, F, Cl, Br, I, NO_2_, and CH_3_). These variations were introduced to evaluate the impact of molecular flexibility, as well as the electronic and steric effects of individual substituents, on enzyme binding affinity and selectivity. To assess the cholinesterase inhibitory activity and structural features of these hybrids, we employed in vitro enzyme inhibition assays, docking studies, and kinetic analyses.

In addition, due to the growing problem of antimicrobial resistance and the urgent need for novel antibacterial agents [50], the synthesized compounds were also evaluated for their antimicrobial potential. Given that coumarin, isatin, and triazole derivatives have demonstrated notable antimicrobial activity in previous studies, the hybrid molecules were screened against a panel of clinically relevant bacterial and fungal strains [51,52].

## 2. Results and Discussion

### 2.1. General Procedure for the Synthesis of Coumarin–Triazole–Isatin Hybrids

In this study, we synthesized 21 coumarin–triazole–isatin hybrids, divided into three subsets based on the linker length between the 4-hydroxycoumarin and triazole moieties. The linker lengths were varied to explore their impact on molecular flexibility, as an extended linker allows for greater conformational flexibility, potentially optimizing interactions within the enzyme’s active site [53,54,55]. The selection of substituents at the 5-position of the isatin ring was guided by their diverse steric and electronic characteristics, allowing a systematic investigation of their influence on ChEs inhibition. This strategy enabled evaluation of how electron-withdrawing or -donating effects, as well as steric bulk, modulate binding interactions within the active sites of both enzymes.

**Scheme 1:**
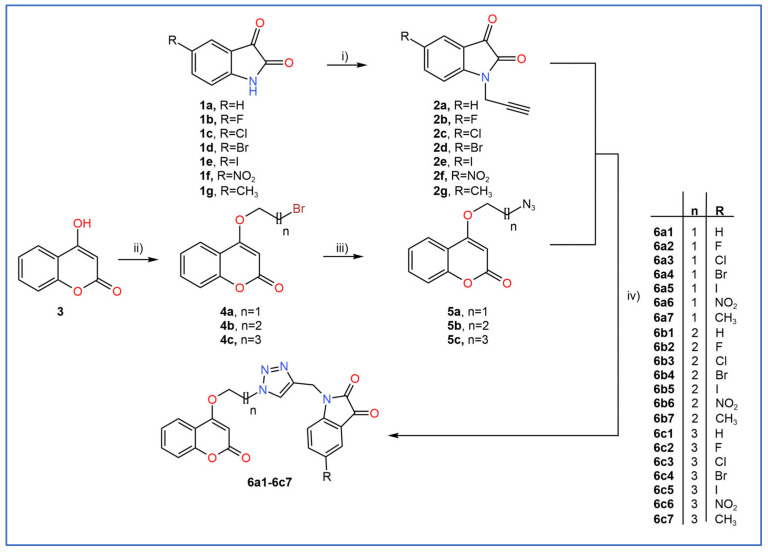
Synthesis of coumarin–triazole–isatin hybrid molecules. Reagents and conditions: (i) propargyl bromide, K_2_CO_3_, DMF, 4 h, RT; (ii) dibromo alkane, K_2_CO_3_, DMF, 6 h, RT; (iii) NaN_3_, DMF, 4 h, RT; (iv) CuSO_4_, sodium ascorbate, DMF, distilled water, 0.5–2 h, RT.

The target compounds (**6a1**–**6c7**) were obtained via a multi-step synthesis, as illustrated in Scheme 1. Initially, isatins were propargylated via an S_N_2 alkylation in dimethylformamide (DMF) using potassium carbonate (K_2_CO_3_) as a base, following established protocols [56,57]. The reactions were carried out at room temperature (RT) for 4 h, affording the corresponding *N*-propargyl isatin derivatives (1-(prop-2-ynyl)indoline-2,3-diones, **2a**–**2g**) in yields exciding 60% (Scheme S1). However, the 5-nitro-substituted derivative was isolated in a significantly lower yield (29%), likely due to the strong electron-withdrawing nature of the nitro group, which weakens the nucleophilicity of the isatin nitrogen and slows down the reaction rate. Attempts to increase the reaction temperature resulted in the formation of undesired byproducts, further limiting the efficiency of this step [57].

*O*-bromoalkyl coumarin intermediates (4-(ω-bromoalkoxy)chromen-2-ones, **4a**–**4c**) were synthesized by reacting 4-hydroxycoumarin with various dibromo alkanes (Br–(CH_2_)ₙ–Br, *n* = 2–4) in DMF using K_2_CO_3_ as a base, enabling systematic variation in the linker length in final products [58,59]. The reactions proceeded via an S_N_2 mechanism at RT over 6 h and afforded the desired products in moderate yields (~40%) (Scheme S2). A byproduct, likely formed through bis-alkylation of two coumarin molecules by a single dibromo alkane, was also isolated in almost equimolar amounts. Its dimeric structure was confirmed by MS analysis and supported by characteristic signals in the ^1^H and ^13^C NMR spectra. Subsequently, *O*-bromoalkyl coumarins (4-(ω-azidoalkoxy)chromen-2-ones, **5a**–**5c**) underwent S_N_2 substitution with sodium azide (NaN_3_) in DMF at RT for 4 h, yielding azide-functionalized products with high purity and yields of 74–93% (Scheme S3). The strong nucleophilicity of –N_3_^−^, attributed to its planar structure and high electron density, facilitated efficient halogen displacement [60].

The coumarin–triazole–isatin hybrids were synthesized through a copper(I)-catalyzed azide–alkyne cycloaddition (CuAAC), a typical “click” reaction that efficiently generates 1,2,3-triazole rings (**6a1**–**6c7**) from previously prepared *N*-propargyl isatin derivatives and *O*-azidoalkyl coumarins under mild conditions [61,62]. The reaction selectively produced 1,4-disubstituted 1,2,3-triazoles, owing to the high regioselectivity of Cu(I)-catalyzed processes. To generate copper (I) in situ, anhydrous copper (II) sulfate (CuSO_4_) and sodium ascorbate were employed as a reducing system [63]. These reagents were pre-dissolved in distilled water before addition to the reaction mixture. The reaction was carried out smoothly at RT and was complete within 0.5–2 h, affording the target hybrid molecules in high yields (above 70%) and purities (Scheme S4).

In the ^1^H NMR spectrum of the coumarin–triazole–isatin hybrid (compound **6a1**), the –OCH_2_^−^ group linked to the coumarin core appeared as a triplet at 4.57 ppm (parts per million), while the adjacent –CH_2_^−^ group attached to the triazole ring resonated as a triplet at 4.86 ppm. The –CH_2_^−^ group connecting the triazole and isatin moieties gave a singlet at 4.96 ppm. The C3-H proton of the coumarin system, due to its position within the aromatic scaffold, appeared as a singlet at 5.91 ppm. Signals corresponding to aromatic protons from isatin and coumarin cores were observed in the 7.04–7.65 ppm range, while proton from triazole ring can be seen as a singlet at 8.34 ppm (when DMSO-*d_6_* is used as a solvent). In the ^13^C NMR spectrum, the aliphatic carbons linking the coumarin and triazole moieties were observed at 35.49 and 68.18 ppm (–OCH_2_CH_2_^−^), while the linker carbon between triazole and isatin heterocycles was detected at 48.89 ppm (–CH_2_^−^). The characteristic signal at 91.52 ppm corresponds to the C-3 carbon of the coumarin ring, while the isatin carbonyl (C=O) resonance appeared downfield at 183.52 ppm. These, together with multiple aromatic and heteroaromatic signals observed between 111.52 and 164.63 ppm, confirm successful hybrid formation. The complete set of ^1^H and ^13^C NMR spectra for all synthesized hybrid compounds is available in the Supplementary Materials (Figures S1–S21).

### 2.2. In Vitro Enzyme Inhibition Assays and Enzyme Kinetics

All synthesized compounds were initially evaluated for their inhibitory activity against AChE and BChE at a concentration of 20 µM, using a modified Ellman’s method [64], with donepezil as a reference compound for comparison. *Electrophorus electricus* AChE (eeAChE) and equine serum BChE (eqBuChE) were selected as enzyme sources due to their high structural and functional similarity to the corresponding human enzymes. eeAChE shares approximately 90% sequence homology with human AChE [65], while eqBuChE shows 90% sequence identity, with all fourteen key active site residues fully conserved [66]. The bioassay results indicated that nearly half of the synthesized molecules exhibited BChE inhibition, with maximum inhibition reaching up to 85%, whereas AChE inhibition was generally modest to moderate, with just a few compounds showing inhibition levels around 60–70%. IC_50_ values were subsequently determined at seven concentration levels (Figures S22 and S23) for compounds that exhibited greater than 50% inhibition at the initial screening concentration, as shown in Table 1.

#### 2.2.1. In Vitro Butyrylcholinesterase Inhibition Assay and SAR Analysis

The synthesized coumarin–triazole–isatin hybrids exhibited considerable affinity toward BChE, although their inhibitory potencies varied markedly across the series, thereby highlighting the influence of structural modifications on enzyme interaction. A well-defined structure–activity relationship (SAR) was observed regarding linker length. Compounds with a four-carbon (butyl) linker generally showed enhanced activity when compared to their counterparts with shorter linkers. Within this subset, compound **6c1** emerged as the most potent inhibitor, exhibiting an IC_50_ value of 1.74 ± 0.29 µM—considerably lower than that of the reference inhibitor donepezil (IC_50_ = 5.24 ± 1.16 µM) under the same experimental conditions. In comparison, compounds with a three-carbon linker showed good to moderate activity, with **6b1** and **6b2** exhibiting IC_50_ values of 8.52 ± 1.28 and 5.90 ± 1.09 µM, respectively. The two-carbon linker series was generally less effective; only **6a1** and **6a2** showed moderate inhibition (IC_50_ = 6.08 ± 1.19 and 9.16 ± 1.69 µM), while the others displayed weak activity. These findings suggest that an extended linker improves molecular flexibility, allowing more favorable alignment and interactions within the spacious active site gorge of BChE.

Substituent variation at the C-5 position of the isatin core played a key role in modulating BChE inhibitory activity. Across all linker series, the most potent compounds featured an unsubstituted isatin moiety, with compound **6c1** showing the lowest IC_50_ = 1.74 ± 0.29 µM as discussed above. This suggests that the absence of a substituent at this position allows for optimal fitting within the enzyme’s active-site gorge, likely due to reduced steric hindrance. Halogen-substituted derivatives demonstrated a structure–activity trend related to substituent size. Fluorine-substituted compounds (**6a2**, **6b2** and **6c2**) exhibited good-to-moderate BChE inhibitory activity, with IC_50_ values ranging from 5.51 to 9.16 µM while chlorine-substituted active derivatives showed slightly weaker activity, with IC_50_ values of 10.58 ± 1.13 µM (**6a3**) and 11.25 ± 2.13 µM (**6b3**). However, the chlorine-substituted compound with the four-carbon linker (**6c3**) demonstrated very low enzyme inhibition (% of inhibition = 5.19 ± 1.14%), suggesting that the increased linker length does not offset the steric hindrance imposed by the larger halogen. It is evident that activity declined markedly with larger halogens such as bromine (**6a4**, **6b4** and **6c4**) and iodine (**6a5**, **6b5** and **6c5**), likely due to steric interference with proper orientation or access to the binding cavity. Nitro-substituted analogs (**6a6**, **6b6** and **6c6**) were the least active, regardless of linker length (% of inhibition < 6%). The strong electron-withdrawing nature and high polarity of the NO_2_ group may alter electron distribution in the isatin scaffold or reduce binding compatibility with the largely hydrophobic active site. Even methyl-substituted derivatives (**6a7**, **6b7** and **6c7**), bearing a weak electron-donating and sterically small group, exhibited reduced potency (% inhibition < 11%), suggesting that even subtle modifications at this position can adversely affect binding. Altogether, these findings emphasize the balance of steric and electronic factors at the C-5 position of isatin, highlighting the importance of minimal substitution to retain or improve BChE inhibitory potency.

#### 2.2.2. In Vitro Acetylcholinesterase Inhibition Assay and SAR Analysis

In contrast to their generally higher activity against BChE, most coumarin–triazole–isatin hybrid compounds exhibited only weak to moderate inhibition of AChE, indicating a degree of selectivity toward the former enzyme (Table 1). Three compounds surpassed the 50% inhibition at threshold concentration level of 20 µM that were further evaluated for their IC_50_ values. **6c1** showed the highest potency with an IC_50_ value of 15.28 ± 1.22 µM, followed by **6b2** and **6b1**, with IC_50_ values of 17.13 ± 1.65 µM and 18.16 ± 1.19 µM, respectively. All three active compounds share structural features that appear favorable for AChE binding: an unsubstituted or small fluorine-substituted isatin core and a medium-to-long alkyl linker (propyl or butyl), offering a balance between conformational flexibility and appropriate spatial orientation within the AChE gorge.

In general, shorter linkers proved as less effective, as most compounds in this group failed to achieve significant AChE inhibition. This may be attributed to insufficient flexibility preventing optimal interactions with key residues in the narrow and aromatic-rich active-site gorge of AChE. Similarly, substitution at the 5-position of the isatin ring with bulky or strongly electron-withdrawing groups (e.g., Br, I, NO_2_) resulted in poor activity, likely due to steric hindrance and disruption of favorable binding interactions. The narrowness and aromatic amino acid residues of AChE’s active site likely impose tighter structural constraints, limiting the number of hybrid molecules that can effectively engage key interactions [67,68].

Altogether, the modest AChE inhibition across the series, compared with stronger BChE activity, highlights a clear preference of the coumarin–triazole–isatin scaffold toward BChE over AChE. This selectivity (Table 1) aligns with the design rationale and supports the development of this scaffold as a promising chemotype for selective BChE inhibition—an increasingly relevant strategy in the treatment of late-stage AD.

#### 2.2.3. Enzyme Kinetics

**Figure 1:**
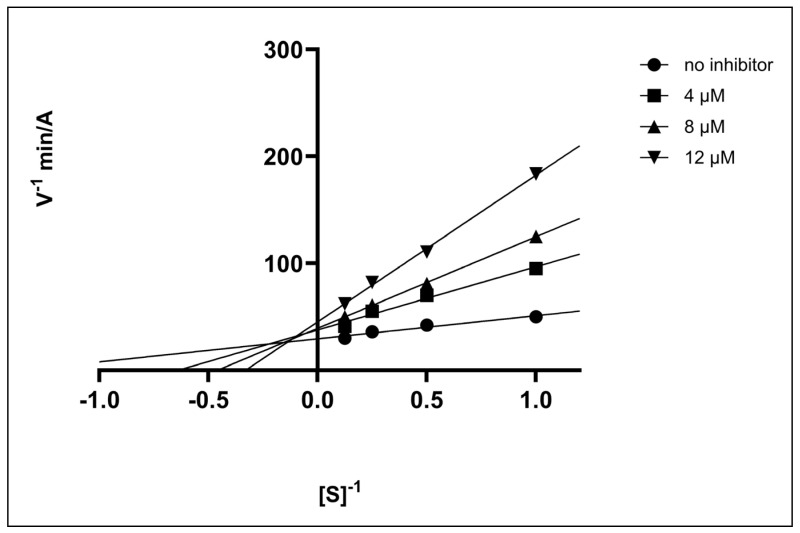
Lineweaver–Burk plot showing mixed inhibition of BChE for compound **6c1**.

To gain further insight into the inhibition mechanism, the most potent BChE inhibitor, compound **6c1** was selected for additional evaluation to determine the type of inhibition. The concentrations of substrate and inhibitor used in the assay are given in Section 3.3.1. Based on the experimental data, a Lineweaver–Burk plot was constructed, indicating a mixed-type inhibition pattern, where K_m_, V_max_, and the slope of the line are all affected by the presence of the inhibitor (Figure 1).

The plot showed an increase in both K_m_ and the slope (K_m_/V_max_), alongside a reduction in V_max_. Moreover, the intersection of the regression lines to the left of the *y*-axis and above the *x*-axis suggests an α value (affinity constant) greater than 1. This observation clearly indicates that compound **6c1** preferentially binds to the free enzyme rather than the enzyme–substrate complex [69].

### 2.3. Evaluation of Antimicrobial Activity

The initial antimicrobial screening using the agar well diffusion method revealed that the coumarin–triazole–isatin hybrids exhibited selective activity against Gram-positive bacteria, while Gram-negative and fungal species remained largely resistant. Only a limited number of compounds demonstrated measurable inhibition zones against *Staphylococcus aureus* ATCC 25923 and *Streptococcus epidermidis* ATCC 12228. Among them, compound 2D showed the highest activity, with zones of inhibition (ZOIs) of 20 mm and 14 mm, respectively, as presented in Table 2.

A preliminary SAR was observed, particularly for *S. aureus*. All derivatives bearing a bromine atom at the C-5 position (**6a4**, **6b4** and **6c4**) of the isatin ring displayed moderate to weak activity (ZOI range 12–20 mm) compared to the reference antibiotic ciprofloxacin (ZOI = 27 mm). In the series with a two-carbon linker (ethyl), only the bromo-substituted compound **6a4** showed activity, while in the three-carbon linker series (propyl), derivative bearing bromine (**6b4**) exhibited moderate antibacterial effects (highest of all synthesized hybrids), while fluorine (**6b2**) and chlorine (**6b3**) substituted derivatives showed only modest antibacterial effect. Similarly, compounds with a four-carbon linker (butyl), including the unsubstituted derivative (**6c1**) as well as those substituted with fluorine (**6c2**), chlorine (**6c3**), or bromine (**6c4**), exhibited only weak antibacterial activity. Compounds substituted with iodine (**6a5**, **6b5** and **6c5**), nitro (**6a6**, **6b6** and **6c6**) or methyl groups (**6a7**, **6b7** and **6c7**) were largely inactive across all linker series; similarly, the unsubstituted analogs **6a1**, and **6a2** also failed to exhibit significant activity. These results suggest that both the nature of the substituent and the linker length influence antibacterial activity.

The minimum inhibitory concentrations (MICs) of compounds **6a4** and **6b4** were determined against *S. aureus* ATCC 25923 and *S. epidermidis* ATCC 12228 using the agar dilution method. Compound **6b4** exhibited MIC values of 6.25 μg/mL and 25 μg/mL against *S. aureus* and *S. epidermidis*, respectively, while compound **6a4** showed an MIC of 12.5 μg/mL against *S. aureus*. In comparison, ciprofloxacin, used as the reference drug, demonstrated MIC values of 0.78 μg/mL for both strains. These results indicate moderate antibacterial activity of the tested hybrids.

The absence of activity against Gram-negative bacteria may be attributed to the structural complexity of their outer membrane, which hinders the diffusion of lipophilic agents and limits access through narrow, selective porin channels [70]. In contrast, Gram-positive bacteria lack this outer membrane, and their porous peptidoglycan layer facilitates passive diffusion of small lipophilic molecules [71]. Accordingly, bromine substitution, optimal linker length, and molar mass may enhance lipophilicity and permeability, contributing to more effective antibacterial action in Gram-positive strains.

### 2.4. Molecular Docking Studies

The binding potential of the designed compounds was assessed through molecular docking simulations targeting the active sites of *Tetronarce californica* AChE (*Tc*AChE) and human BChE (hBChE). The evaluation was based on the nature and strength of binding interactions, as well as the calculated scores using the ChemGauss4 scoring function, which estimates ligand–protein interaction strength based on Gaussian shape-fitting algorithms and chemical complementarity. Lower ChemGauss4 scores indicate better binding affinity, with more negative values corresponding to stronger predicted interactions [72]. The binding interactions of the most active hybrid compound were compared with donepezil, which was used as a reference in in vitro biological assays (Table S1).

While eeAChE and eqBChE were used in enzymatic assays, *Tc*AChE and hBChE were selected for docking due to their well-resolved crystal structures. *Tc*AChE, which shares high sequence and structural similarity with hAChE, serves as a reliable surrogate in computational studies, while docking to hBChE provides a direct model for the human target [65,66].

#### 2.4.1. Molecular Docking of Most Active Compound into BChE

**Figure 2:**
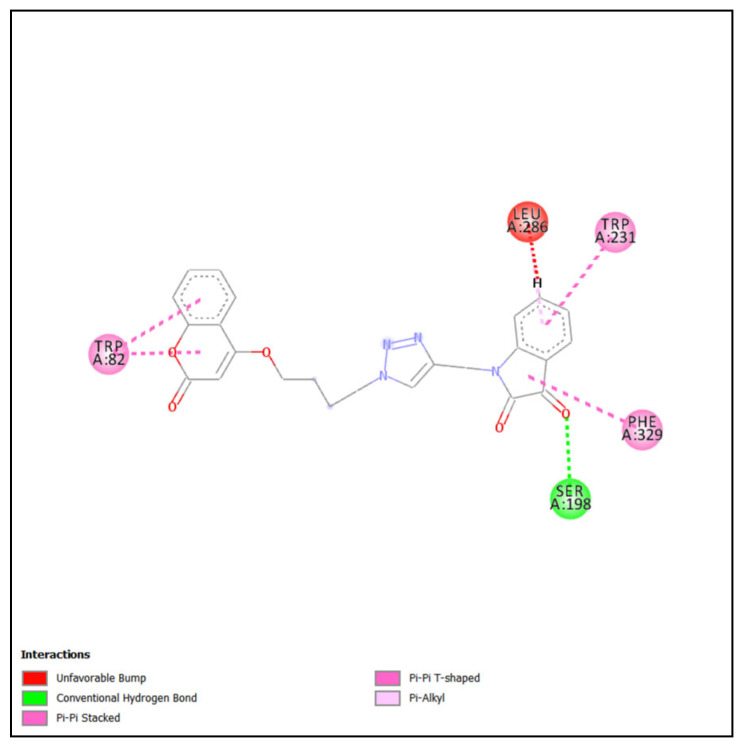
Two-dimensional interaction diagram of **6c1** docked into the active site of hBChE (PDB ID: 5K5E). Color coding represents distinct interaction types: green—hydrogen bonds; purple—π–π stacking; orange—π–alkyl interactions; and red—steric clashes.

The high-resolution X-ray crystal structure of BChE in complex with 6QS (PDB ID: 5K5E, resolution 2.7 Å) was selected for performing the docking studies. Compound **6c1** was docked into the active site of hBChE, and the highest-ranked docking poses were then analyzed to identify key interactions (Figure 2). The coumarin moiety engaged in a π–π stacking interaction with Trp82 (CAS). Moreover, Phe329 (PAS) formed a π–alkyl interaction with the isatin core, while Ser198 (CAS) established a hydrogen bond (H-bond) with the carbonyl oxygen at position 3 of the ring. Trp231 (PAS) was involved in a π–π T-shaped interaction with the aromatic ring of the isatin heterocycle. Although a steric clash with Leu286 from the acyl binding pocket was observed, its impact on activity appears negligible, likely due to the inherent flexibility of the enzyme’s active site, allowing necessary conformational adjustments. These findings indicate that the coumarin moiety of the hybrid is primarily associated with interactions within the CAS, while the isatin heterocycle engages in interactions both within the CAS and PAS. To the best of our knowledge, such dual-site engagement by an isatin-based moiety has not been previously reported in the context of cholinesterase inhibitors, suggesting a potentially novel mode of binding.

**Figure 3:**
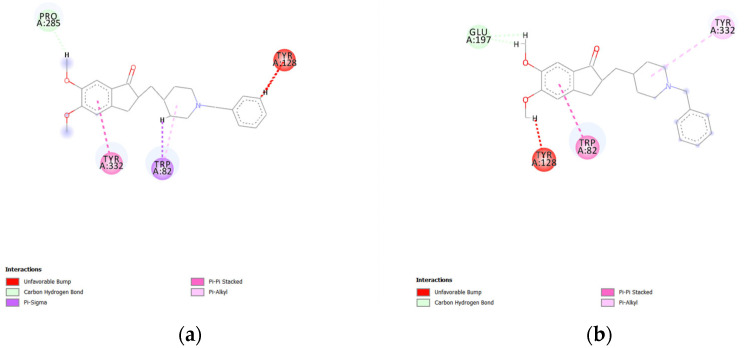
Two-dimensional interaction diagram of: (**a**) *S*-donepezil and (**b**) *R*-donepezil docked into the active site of hBChE (PDB ID: 5K5E). Colour coding represents distinct interaction types: green—hydrogen bonds, purple—π–π stacking, orange—π-alkyl interactions and red—steric clashes.

The results of molecular docking studies of the *R*- and *S*-enantiomers of donepezil revealed distinct differences in their binding orientations and interactions (Figure 3). The *R*-enantiomer’s piperidine moiety established π–σ and π–alkyl interactions with Trp82. Furthermore, Tyr332 (PAS) formed a π–π stacked interaction with the benzene ring of the indanone moiety. Nevertheless, the benzene ring of the *R*- enantiomer exhibited an unfavorable steric clash with Tyr128 (PAS), which may impact the overall binding affinity and inhibitory activity. The molecular docking of *S*- donepezil disclosed a π–π stacked interaction between Trp82 and the benzene ring of the indanone moiety. Also, Tyr332 was involved in a π–alkyl interaction with the piperidine ring, contributing to ligand stabilization. Though, an unfavorable steric clash between the methoxy group attached to the indanone and Tyr128 may influence the binding affinity.

Molecular docking results suggest that the coumarin–triazole–isatin hybrid exhibits superior binding interactions within the BChE active site compared to both enantiomers of donepezil, beside similar ChemGauss4 scores. The combination of diverse binding interactions and a well-accommodated structure within the active site suggests that the hybrid could exhibit enhanced inhibitory activity against BChE as supported by our in vitro inhibition studies. Conversely, compounds **6c4**, **6c5**, **6c6**, and **6c7**—despite belonging to the same linker-length series as the most potent inhibitor **6c1**—failed to establish key binding interactions within the BChE active site. In particular, H-bond with Ser198 was not observed in all four compounds, while hydrophobic interactions with Trp82 were not detected for **6c4** and **6c5** (Figure S24). The lack of these critical interactions likely compromises ligand stabilization within the active site, thereby contributing to their diminished inhibitory activity [68,73,74].

#### 2.4.2. Molecular Docking of Most Active Compound into AChE

**Figure 4:**
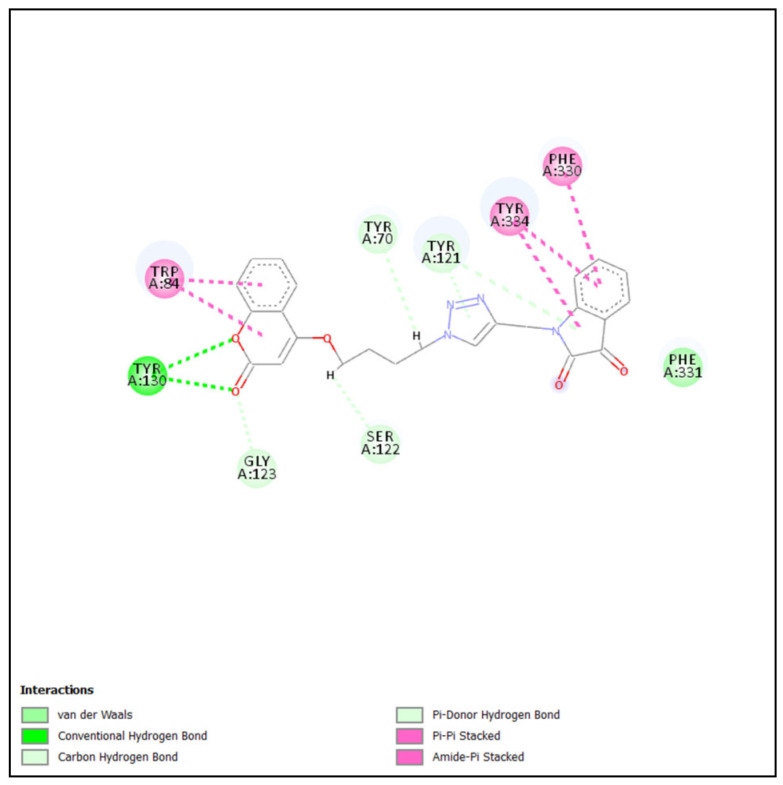
Two-dimensional interaction diagram of **6c1** docked into the active site of *Tc*BChE (PDB ID: 5NAP). Color coding represents distinct interaction types: green—hydrogen bonds, purple—π–π stacking, orange—π-alkyl interactions and red—steric clashes.

The X-ray crystallographic structure of *Tc*AChE complexed with a non-chiral donepezil-like inhibitor 17 (PDB ID: 5NAP, resolution 2.17 Å) was used. Compound **6c1** was docked into the binding site of the reference ligand, and its top-ranked poses were analyzed to visualize key interactions (Figure 4). The coumarin moiety established a π–π stacking interaction with Trp84 from CAS, alongside a H-bond with Tyr130 (CAS). Additionally, Phe330 from the aromatic cluster near the CAS engaged in π–π stacking with the benzene ring of the isatin core, while Tyr334 from PAS participated in both π–π stacking with the same benzene ring and an amide–π interaction with the 2,3-diketone moiety of the isatin scaffold.

**Figure 5:**
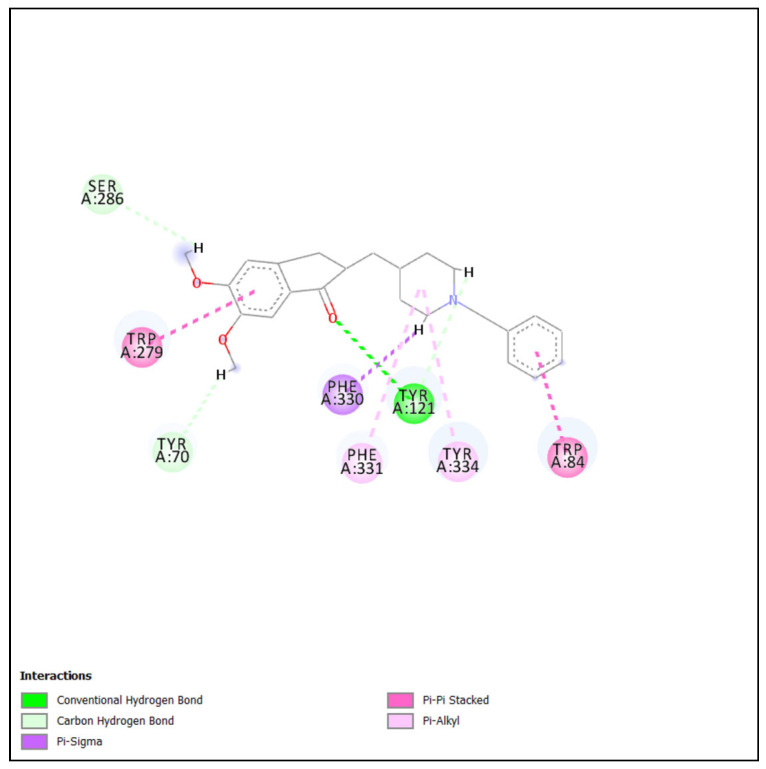
Two-dimensional interaction diagram of *S*-donepezil docked into the active site of *Tc*BChE (PDB ID: 5NAP). Color coding represents distinct interaction types: green—hydrogen bonds, purple—π–π stacking, orange—π–alkyl interactions and red—steric clashes.

Docking results of *S*-donepezil (more active enantiomer towards AChE) revealed that the benzene ring of the indanone moiety established π–π stacking interactions with Trp279 (PAS) of *Tc*AChE. Moreover, the carbonyl oxygen of the indanone engaged in H-bonding with Tyr121 (PAS), further stabilizing the binding at the peripheral site. The piperidine ring formed π–alkyl interactions with Phe331 (PAS) and Tyr334, as well as a π–σ interaction with Phe330. The benzene ring exhibited π–π stacking interactions with Trp84, strengthening its interactions within the catalytic active site. These interactions are illustrated in Figure 5.

The best-scoring pose of *S*-donepezil (−17.26) reflects a stronger predicted binding interaction than that of compound 6c1 (−15.44), in agreement with the observed inhibitory activity. Additionally, it forms strong π–π stacking interactions with Trp279, stabilizing its binding and potentially limiting substrate access, while H-bond with Tyr121 enhancing its affinity. In contrast, **6c1** lacks interaction with Trp279 and Tyr121, instead relying primarily on H- bonding with Tyr130. For comparison, the least active compounds (**6a3**, **6a4**, **6a5**, **6a6**, and **6a7**), all belonging to the shortest-linker series (ethyl), lacked the key binding interactions observed in potent inhibitors—specifically, no hydrophobic stacking with Trp84 and no H-bonding with Tyr121 were detected in their docking poses (Figure S25). These findings highlight the pivotal role of those interactions in achieving strong inhibitory activity within the AChE active site [68,75].

## 3. Materials and Methods

### 3.1. Chemicals and Instruments

All chemicals, reagents, and solvents used for synthesis were obtained from Sigma-Aldrich (St. Louis, MO, USA), Thermo Scientific (Waltham, MA, USA), Acros Organics (Geel, Belgium), BLDpharm (Shanghai, China), Alkaloid AD (Skopje, N. Macedonia), and VWR Chemicals BDH (VWR International—Radnor, PA, USA), and were used as received without further purification, unless otherwise stated. Reaction progress was monitored by thin-layer chromatography (TLC) performed on pre-coated silica gel 60 GF_254_ plates (Merck, Darmstadt, Germany). All reported yields refer to isolated products after purification performed by column chromatography on silica gel 60 (0.063–0.2 mm) (Merck, Darmstadt, Germany). Melting points were determined using a Boetius PHMK 05 apparatus (VEB Wagetechnik Rapido, Radebeul, Germany). Infrared (IR) spectra were recorded on a Cary 630 FT-IR spectrometer fitted with a diamond attenuated total reflection (ATR) module (Agilent Technologies, Santa Clara, CA, USA). NMR spectra were acquired on a Bruker Ascend 400 (400 MHz) spectrometer (Bruker, Billerica, MA, USA) and were measured in DMSO-*d_6_* or CDCl_3_ (Sigma-Aldrich, St. Louis, MO, USA). In ^1^H NMR spectra, chemical shifts are reported in parts per million (ppm) downfield from tetramethylsilane (TMS) as an internal standard, with integration values, multiplicities (s-singlet; d-doublet; t-triplet; m-multiplet) and coupling constants (*J*) in Hertz (Hz). The mass spectra were recorded on a TSQ Quantum Access MAX triple quadrupole mass spectrometer equipped with an electrospray ionization (ESI) source (Thermo Fisher Scientific, Waltham, MA, USA) operated in the positive ion mode, while determination of exact masses was performed using an Agilent 6546 Q-TOF high-resolution mass spectrometer (Agilent Technologies, Santa Clara, CA, USA).

### 3.2. Synthetic Procedures

#### 3.2.1. Synthesis of 1-(Prop-2-ynyl)indoline-2,3-diones (**2a**–**2g**)

Isatin or its 5-substituted derivative (**1a**–**1g**) (20 mmol, 1 equiv.) was dissolved in anhydrous DMF (35 mL), followed by consecutive addition of anhydrous K_2_CO_3_ (30 mmol, 1.5 equiv.) and propargyl bromide (30 mmol, 1.5 equiv., added dropwise). The reaction mixture was stirred at RT until completion, as monitored by TLC. Upon completion, it was poured onto crushed ice and left to stand until the ice had completely melted. The resulting precipitate was collected by filtration, thoroughly washed with ice-cold water, and dried in vacuo. Purification by column chromatography (hexane/ethyl acetate, 7:3, *v*/*v*) afforded pure 1-(prop-2-ynyl)indoline-2,3-dione or its 5-substituted derivatives in satisfactory yields (**2a**–**2g**).

#### 3.2.2. Synthesis of 4-(ω-bromoalkoxy)chromen-2-ones (**4a**–**4c**)

4-hydroxycoumarin (**3**) (31 mmol, 1 equiv.) was dissolved in anhydrous DMF (70 mL), followed by addition of anhydrous K_2_CO_3_ (46 mmol, 1.5 equiv.). The resulting suspension was stirred at RT for 15 min, after which 1,2-dibromoethane, 1,3-dibromopropane, or 1,4-dibromobutane (37 mmol, 1.2 equiv.) was added. The reaction mixture was continuously stirred at RT until completion, as monitored by TLC. It was then poured onto crushed ice, and the precipitate that formed was collected by filtration, washed with ice-cold water, dried under reduced pressure, and purified by column chromatography (hexane/ethyl acetate, 6:4 *v*/*v*) to afford the corresponding 4-(ω-bromoalkoxy)chromen-2-ones(**4a**–**4c**) in good yields.

#### 3.2.3. Synthesis of 4-(ω-azidoalkoxy)chromen-2-ones (**5a**–**5c**)

A solution of the corresponding 4-(ω-bromoalkoxy)chromen-2-one (**4a**–**4c**) (15 mmol, 1 equiv.) in anhydrous DMF (20 mL) was prepared, and NaN_3_ (16.5 mmol, 1.1 equiv.) was added. The reaction mixture was stirred at RT until completion, as monitored by TLC. Upon completion, it was poured onto crushed ice and left to stand until the ice had fully melted. The precipitate formed was collected by filtration, washed with ice-cold water, and dried in vacuo. Purification was achieved by column chromatography using a hexane/ethyl acetate (7:3 *v*/*v*) as the eluent to obtain pure 4-(ω-azidoalkoxy)chromen-2-one (**5a**–**5c**) in high yields.

#### 3.2.4. Synthesis of Coumarin–Triazole–Isatin Hybrids (**6a1**–**6c7**)

Equimolar amounts of 1-(prop-2-ynyl)indoline-2,3-dione or its 5-substituted derivative (**2a**–**2g**) (3 mmol) and 4-(ω-azidoalkoxy)chromen-2-one (**5a**–**5c**) (3 mmol) were dissolved in anhydrous DMF (6 mL). Freshly prepared aqueous solutions of CuSO_4_ (0.3 mL, 1 M) and sodium ascorbate (0.3 mL, 2 M) were added, yielding a DMF-to-water ratio of 10:1 in the reaction mixture. The reaction was stirred at RT until completion, as monitored by TLC. Upon completion, the mixture was poured onto crushed ice and left to stand until fully melted. The resulting precipitate was collected by filtration, thoroughly washed with ice-cold water to remove residual copper and ascorbate, dried in vacuo, and purified by column chromatography (hexane/ethyl acetate, 7:3 → 2.5:7.5 *v*/*v*), affording the desired coumarin–triazole–isatin hybrids (**6a1**–**6c7**) as solids in excellent yields. The characterization data for all derivatives are presented below.

1-((1-(2-((2-oxo-2*H*-chromen-4-yl)oxy)ethyl)-1*H*-1,2,3-triazol-4-yl)methyl)indoline-2,3-dione (**6a1**).

Yield: 79%. Light-orange solid. Melting point: 216.9–218.2 °C. FT-IR (ATR) ν max (cm^−1^): 749, 1247, 1375, 1463, 1607, 1733, 2923, and 3133. ^1^H NMR (400 MHz, DMSO-*d_6_*) δ (ppm): 4.57 (2H, t, *J* = 4.8 Hz, CH_2_), 4.86 (2H, t, *J* = 4.8 Hz, CH_2_), 4.96 (2H, s, CH_2_), 5.91 (1H, s, CH), 7.04–7.10 (2H, m, ArH), 7.26 (1H, t, *J* = 7.6 Hz, ArH), 7.37 (1H, d, *J* = 8.4 Hz, ArH), 7.46–7.49 (2H, m, ArH), 7.51–7.65 (2H, m, ArH), 8.34 (1H, s, ArH) (Figure S1a). ^13^C NMR (100 MHz, DMSO-*d_6_*) δ (ppm): 35.49, 48.89, 68.18, 91.52, 111.52, 115.30, 116.86, 117.98, 123.16, 123.78, 124.65, 124.90, 124.93, 133.27, 138.39, 142.00, 150.58, 153.14, 158.21, 161.84, 164.63, 183.52 (Figure S1b). *m*/*z* = 417.2 [M+H]^+^, 199.12, 158.18, 198.10, 146.20, 318.06, and 172.15. HRMS (ESI^+^): *m*/*z* calcd. for C_22_H_17_N_4_O_5_ [M+H]^+^: 417.11990; found: 417.11949.

5-fluoro-1-((1-(2-((2-oxo-2*H*-chromen-4-yl)oxy)ethyl)-1*H*-1,2,3-triazol-4-yl)methyl)indoline-2,3-dione (**6a2**).

Yield: 71%. Orange solid. Melting point: 240.5–241.2 °C. FT-IR (ATR) ν max (cm^−1^): 1247, 1377, 1479, 1618, 1733, 2922, and 3133. ^1^NMR (400 MHz, DMSO-*d_6_*) δ (ppm): 4.57 (2H, t, *J* = 4.8 Hz, CH_2_), 4.86 (2H, t, *J* = 4.8 Hz, CH_2_), 4.96 (2H, s, CH_2_), 5.91 (1H, s, CH), 7.07–7.10 (1H, m, ArH), 7.24 (1H, t, *J* = 7.6 Hz, ArH), 7.29–7.37 (2H, m, ArH), 7.42–7.44 (1H, m, ArH), 7.58–7.65 (2H, m, ArH), 8.34 (1H, s, ArH) (Figure S2a). ^13^C NMR (100 MHz, DMSO-*d_6_*) δ (ppm): 35.57, 48.92, 68.21, 91.49, 111.78, 112.02 (m), 112.87 (m), 112.94 (m), 115.27, 116.83, 118.87 (m), 118.94 (m), 123.17, 124.14 (m), 124.38 (m), 124.61 (m), 124.96, 133.25, 141.91, 146.75, 153.12, 157.71, 158.23, 161.84, 164.63, 182.91 (Figure S2b). *m*/*z* = 435.1 [M+H]^+^, 217.06, 176.07, 418.07, 164.14, 198.10, and 336.03. HRMS (ESI^+^): *m*/*z* calcd. for C_22_H_16_FN_4_O_5_ [M+H]^+^: 435.11047; found: 435.11021.

5-chloro-1-((1-(2-((2-oxo-2*H*-chromen-4-yl)oxy)ethyl)-1*H*-1,2,3-triazol-4-yl)methyl)indoline-2,3-dione (**6a3**).

Yield: 74%. Orange solid. Melting point: 267.1–267.7 °C. FT-IR (ATR) ν max (cm^−1^): 749, 1247, 1375, 1463, 1604, 1733, 2923, and 3132. ^1^NMR (400 MHz, DMSO-*d_6_*) δ (ppm): 4.56 (2H, t, *J* = 4.8 Hz, CH_2_), 4.86 (2H, t, *J* = 4.8 Hz, CH_2_), 4.96 (2H, s, CH_2_), 5.91 (1H, s, CH), 7.09 (1H, d, *J* = 8.4 Hz, ArH), 7.23 (1H, t, *J* = 7.6 Hz, ArH), 7.37 (1H, d, *J* = 8.4 Hz, ArH), 7.46–7.49 (1H, m, ArH), 7.57–7.65 (3H, m, ArH), 8.34 (1H, s, ArH) (Figure S3a). ^13^C NMR (100 MHz, DMSO-*d_6_*) δ (ppm): 35.48, 48.89, 68.18, 91.52, 111.51, 115.30, 116.86, 117.98, 123.16, 123.78, 124.65, 124.90, 124.93, 133.27, 138.39, 142.00, 150.58, 153.13, 158.21, 161.84, 164.63, 183.53 (Figure S3b). *m*/*z* = 452.2 [M+H]^+^, 435.09, 217.12, 176.05, 164.09, 216.03, and 198.04. HRMS (ESI^+^):*m*/*z* calcd. for C_22_H_16_ClN_4_O_5_ [M+H]^+^: 451.08092; found: 451.08031.

5-bromo-1-((1-(2-((2-oxo-2*H*-chromen-4-yl)oxy)ethyl)-1*H*-1,2,3-triazol-4-yl)methyl)indoline-2,3-dione (**6a4**).

Yield: 83%. Orange solid. Melting point: 268.8–270.3 °C. FT-IR (ATR) ν_max (cm^−1^): 773, 1243, 1377, 1437, 1603, 1720, 2922, and 3068. ^1^NMR (400 MHz, DMSO-*d_6_*) δ (ppm): 4.63 (2H, t, *J* = 4.6 Hz, CH_2_), 4.93 (2H, t, *J* = 4.8 Hz, CH_2_), 5.03 (2H, s, CH_2_), 5.98 (1H, s, CH), 7.11 (1H, d, *J* = 8.0 Hz, ArH), 7.29–7.32 (1H, t, *J* = 7.6 Hz, ArH), 7.44 (1H, d, *J* = 8 Hz, ArH), 7.65–7.75 (4H, m, ArH), 8.41 (1H, s, ArH) (Figure S4a). ^13^C NMR (100 MHz, DMSO-*d_6_*) δ (ppm): 35.59, 48.92, 68.21, 91.49, 113.61, 115.26, 115.57, 116.85, 119.78, 123.16, 124.62, 124.97, 127.13, 133.25, 140.02, 141.82, 149.40, 153.12, 157.81, 161.84, 164.62, 182.27 (Figure S4b). *m*/*z* = 497.1 [M+H]^+^, 237.90, 278.96, 277.89, 397.86, 225.93, and 251.97. HRMS (ESI^+^): *m*/*z* calcd. for C_22_H_16_BrN_4_O_5_ [M+H]^+^: 495.03041; found: 495.03025.

5-iodo-1-((1-(2-((2-oxo-2*H*-chromen-4-yl)oxy)ethyl)-1*H*-1,2,3-triazol-4-yl)methyl)indoline-2,3-dione (**6a5**).

Yield: 71%. Orange solid. Melting point: 266.1–268.6 °C. FT-IR (ATR) ν_max (cm^−1^): 768, 1246, 1375, 1431, 1605, 1710, and 3081. ^1^NMR (400 MHz, DMSO-*d_6_*) δ (ppm): 4.56 (2H, t, *J* = 4.8 Hz, CH_2_), 4.86 (2H, t, *J* = 4.8 Hz, CH_2_), 4.95 (2H, s, CH_2_), 5.91 (1H, s, CH), 6.92 (1H, d, *J* = 8.4 Hz, ArH), 7.24 (1H, t, *J* = 7.6 Hz, ArH), 7.38 (1H, d, *J* = 8.4 Hz, ArH), 7.58–7.66 (2H, m, ArH), 7.75 (2H, t, *J* = 8.4 Hz, ArH), 8.33 (1H, s, ArH) (Figure S5a). ^13^C NMR (100 MHz, DMSO-*d_6_*) δ (ppm): 35.54, 48.91, 68.20, 86.80, 91.49, 113.90, 115.26, 116.86, 120.04, 123.16, 124.64, 124.97, 132.57, 133.27, 145.82, 149.83, 153.12, 157.54, 161.84, 164.62, 182.19 (Figure S5b). *m*/*z* = 543.1 [M+H]^+^, 283.83, 324.84, 443.92, 271.89, 157.05, and 323.79. HRMS (ESI^+^) *m*/*z* calcd. for C_22_H_16_IN_4_O_5_ [M+H]^+^: 543.01655; found: 543.01609.

5-nitro-1-((1-(2-((2-oxo-2*H*-chromen-4-yl)oxy)ethyl)-1*H*-1,2,3-triazol-4-yl)methyl)indoline-2,3-dione (**6a6**).

Yield: 72%. Light-yellow solid. Melting point: 236.2–238.7 °C. FT-IR (ATR) ν_max (cm^−1^): 1249, 1329, 1455, 1607, 1687, 2923, and 3073. ^1^NMR (400 MHz, DMSO-*d_6_*) δ (ppm): 4.55 (2H, t, *J* = 4.8 Hz, CH_2_), 4.87 (2H, t, *J* = 4.8 Hz, CH_2_), 5.06 (2H, s, CH_2_), 5.89 (1H, s, CH), 7.16–7.23 (2H, m, ArH), 7.33 (1H, t, *J* = 11 Hz ArH), 7.58 (2H, t, *J* = 7.4 Hz, ArH), 8.20 (1H, s, ArH), 8.28 (1H, d, *J* = 8.4 Hz, ArH), 8.36 (1H, s, ArH) (Figure S6a). ^13^C NMR (100 MHz, DMSO-*d_6_*) δ (ppm): 35.95, 48.97, 60.21 *, 68.29, 91.45, 111.85, 115.19, 116.77, 118.47, 119.60, 123.18, 124.54, 125.12, 133.01, 133.12, 141.56, 143.44, 153.06, 154.66, 158.78, 161.82, 164.61, 181.31 (Figure S6b). *m*/*z* = 462.1 [M+H]^+^, 300.01, 244.09, 202.95, 241.99, 198.01, and 190.90. HRMS (ESI^+^): *m*/*z* calcd. for C_22_H_16_N_5_O_7_ [M+H]^+^: 462.10498; found: 462.10476.

* 60.21 ppm corresponds to ethyl acetate trace (Figure S6b).

5-methyl-1-((1-(2-((2-oxo-2*H*-chromen-4-yl)oxy)ethyl)-1*H*-1,2,3-triazol-4-yl)methyl)indoline-2,3-dione (**6a7**).

Yield: 79%. Orange solid. Melting point: 247.6–249.4 °C. FT-IR (ATR) ν_max (cm^−1^): 772, 1243, 1377, 1491, 1619, 1726, 2922, and 3068. ^1^NMR (400 MHz, DMSO-*d_6_*) δ (ppm): 2.20 (3H, s, Ar-CH_3_), 4.56 (2H, t, *J* = 5.0 Hz, CH_2_), 4.86 (2H, t, *J* = 4.8 Hz, CH_2_), 4.93 (2H, s, CH), 5.91 (1H, s, CH_2_), 6.94 (1H, d, *J* = 8 Hz, ArH), 7.24 (2H, t, *J* = 8 Hz, ArH), 7.35 (2H, t, *J* = 9.4 Hz, ArH), 7.58–7.66 (2H, m, ArH), 8.33 (1H, s, ArH) (Figure S7a). ^13^C NMR (100 MHz, DMSO-*d_6_*) δ (ppm): 20.45, 35.49, 48.89, 68.20, 91.47, 111.35, 115.27, 116.81, 117.91, 123.15, 124.62, 124.90, 125.10, 133.13, 133.23, 138.64, 142.04, 148.40, 153.12, 158.23, 161.83, 164.62, 183.74 (Figure S7b). *m*/*z* = 431.1 [M+H]^+^, 172.11, 213.08, 160.15, 332.05, 186.06, and 212.04. HRMS (ESI^+^): *m*/*z* calcd. for C_23_H_19_N_4_O_5_ [M+H]^+^: 431.13555; found: 431.13544.

1-((1-(3-((2-oxo-2*H*-chromen-4-yl)oxy)propyl)-1*H*-1,2,3-triazol-4-yl)methyl)indoline-2,3-dione (**6b1**).

Yield: 92%. Light-orange solid. Melting point: 213.2–214.1 °C. FT-IR (ATR) ν_max (cm^−1^): 749, 1243, 1381, 1470, 1613, 1733, and 3078. ^1^NMR (400 MHz, CDCl_3_) δ (ppm): 2.53–2.59 (2H, m, CH_2_), 4.17 (2H, t, *J* = 5.6 Hz, CH_2_), 4.62 (2H, t, *J* = 6.8 Hz, CH_2_), 5.01 (2H, s, CH_2_), 5.62 (1H, s, CH), 7.10–7.13 (1H, t, *J* = 7.6 Hz ArH), 7.25–7.32 (3H, m, ArH), 7.53–7.61 (3H, m, ArH), 7.74 (2H, d, *J* = 6.4 ArH) (Figure S8a). ^13^C NMR (100 MHz, CDCl_3_) δ (ppm): 29.15, 35.37, 47.11, 65.64, 90.96, 111.39, 115.32, 116.94, 117.52, 122.68, 123.36, 124.00, 124.13, 125.38, 132.65, 138.66, 150.14, 153.33, 158.00, 162.47, 165.00, 183.00 (Figure S8b). *m*/*z* = 431.1 [M+H]^+^, 269.00, 241.02, 132.14, 157.10, 185.05, and 130.14. HRMS (ESI^+^): *m*/*z* calcd. for C_23_H_19_N_4_O_5_ [M+H]^+^: 431.13555; found: 431.13543.

5-fluoro-1-((1-(3-((2-oxo-2*H*-chromen-4-yl)oxy)propyl)-1*H*-1,2,3-triazol-4-yl)methyl)indoline-2,3-dione (**6b2**).

Yield: 82%. Light-orange solid. Melting point: 205.1–206.6 °C. FT-IR (ATR) ν_max (cm^−1^): 766, 1230, 1379, 1482, 1603, 1727, 2923, and 3068. ^1^NMR (400 MHz, DMSO-*d_6_*) δ (ppm): 2.33–2.39 (2H, m, CH_2_), 4.20 (2H, t, *J* = 6.2 Hz, CH_2_), 4.56 (2H, t, *J* = 6.8 Hz, CH_2_), 4.92 (2H, s, CH_2_), 5.83 (1H, s, CH), 7.13–7.16 (1H, m, ArH), 7.31–7.38 (2H, m, ArH), 7.44–7.49 (2H, m, ArH), 7.61–7.66 (1H, m, ArH), 7.71–7.73 (1H, m, ArH), 8.22 (1H, s, ArH) (Figure S9a). ^13^C NMR (100 MHz, DMSO-*d_6_*) δ (ppm): 29.14, 35.54, 47.14, 67.14, 91.04, 111.80, 112.04 (m), 112.94 (m), 113.01 (m), 115.52, 116.85, 118.94 (m), 123.44, 124.27 (m), 124.36 (m), 124.52 (m), 133.23, 141.78, 146.83, 153.17, 162.00, 165.16, 182.90 (Figure S9b). *m*/*z* = 449.1 [M+H]^+^, 286.97, 258.99, 150.07, 175.14, 203.04, and 148.13. HRMS (ESI^+^): *m*/*z* calcd. for C_23_H_18_FN_4_O_5_ [M+H]^+^: 449.12612; found: 449.12603.

5-chloro-1-((1-(3-((2-oxo-2*H*-chromen-4-yl)oxy)propyl)-1*H*-1,2,3-triazol-4-yl)methyl)indoline-2,3-dione (**6b3**).

Yield: 78%. Orange solid. Melting point: 152.2–154.8 °C. FT-IR (ATR) ν_max (cm^−1^): 767, 1236, 1373, 1465, 1605, 1711, 2923, and 3165. ^1^NMR (400 MHz, DMSO-*d_6_*) δ (ppm): 2.33–2.39 (2H, m, CH_2_), 4.20 (2H, t, *J* = 6.4 Hz, CH_2_), 4.56 (2H, t, *J* = 6.8 Hz, CH_2_), 4.93 (2H, s, CH_2_), 5.84 (1H, s, CH), 7.16 (1H, d, *J* = 8.4 Hz, ArH), 7.31–7.39 (2H, m, ArH), 7.60–7.66 (3H, m, ArH), 7.71–7.73 (1H, m, ArH), 8.22 (1H, s, ArH) (Figure S10a). ^13^C NMR (100 MHz, DMSO-*d_6_*) δ (ppm): 29.15, 35.58, 47.15, 67.15, 91.05, 113.28, 115.52, 116.85, 119.40, 123.44, 124.35, 124.42, 124.53, 128.06, 133.23, 137.32, 141.71, 149.10, 153.17, 157.97, 162.00, 165.16, 182.41(Figure S10b). *m*/*z* = 465.0 [M+H]^+^, 302.93, 274.96, 166.01, 191.00, 218.98, and 164.03. HRMS (ESI^+^): *m*/*z* calcd. for C_23_H_18_ClN_4_O_5_ [M+H]^+^: 465.09657; found: 465.09623.

5-bromo-1-((1-(3-((2-oxo-2*H*-chromen-4-yl)oxy)propyl)-1*H*-1,2,3-triazol-4-yl)methyl)indoline-2,3-dione (**6b4**).

Yield: 90%. Orange solid. Melting point: 225.2–227.2 °C. FT-IR (ATR) ν_max (cm^−1^): 770, 1237, 1374, 1461, 1603, 1702, 2923, and 3144. ^1^NMR (400 MHz, CDCl_3_) δ (ppm): 2.52–2.59 (2H, m, CH_2_), 4.19 (2H, t, *J* = 6.4 Hz, CH_2_), 4.62 (2H, t, *J* = 6.4 Hz, CH_2_), 4.99 (2H, s, CH_2_), 5.62 (1H, s, CH), 7.26–7.32 (3H, m, ArH), 7.56 (1H, t, *J* = 7.8 Hz, ArH), 7.66–7.74 (4H, m, ArH) (Figure S11a). ^13^C NMR (100 MHz, CDCl_3_) δ (ppm): 29.15, 35.53, 47.16, 67.17, 86.80, 91.06, 114.03, 115.53, 116.86, 120.05, 123.44, 124.30, 124.53, 132.59, 133.23, 141.72, 145.93, 149.89, 153.18, 157.53, 162.00, 165.16, 182.18 (Figure S11b). *m*/*z* = 509.0 [M+H]^+^, 346.85, 318.87, 209.89, 234.70, 262.80, and 232.93. HRMS (ESI^+^): *m*/*z* calcd. for C_23_H_18_BrN_4_O_5_ [M+H]^+^: 509.04606; found: 509.04569.

5-iodo-1-((1-(3-((2-oxo-2*H*-chromen-4-yl)oxy)propyl)-1*H*-1,2,3-triazol-4-yl)methyl)indoline-2,3-dione (**6b5**).

Yield: 76%. Orange solid. Melting point: 224.4–227.0 °C. FT-IR (ATR) ν_max (cm^−1^): 770, 1239, 1375, 1460, 1603, 1707, 2921, and 3146. ^1^NMR (400 MHz, DMSO-*d_6_*) δ (ppm): 2.32–2.38 (2H, m, CH_2_), 4.20 (2H, t, *J* = 6.4 Hz, CH_2_), 4.56 (2H, t, *J* = 6.8 Hz, CH_2_), 4.91 (2H, s, CH_2_), 5.84 (1H, s, CH), 6.98 (1H, d, *J* = 8.4 Hz, ArH), 7.31–7.39 (2H, m, ArH), 7.62–7.66 (1H, m, ArH), 7.73 (1H, d, *J* = 8Hz, ArH), 7.80 (1H, s, ArH), 7.90–7.92 (1H, m, ArH), 8.21 (1H, s, ArH) (Figure S12a). ^13^C NMR (100 MHz, DMSO-*d_6_*) δ (ppm): 29.14, 35.87, 47.15, 67.12, 91.03, 111.97, 115.50, 116.85, 118.43, 119.62, 123.43, 124.51, 133.24, 141.42, 143.58, 153.15, 154.76, 158.72, 161.96, 165.14, 181.31 (Figure S12b). *m*/*z* = 557.0 [M+H]^+^, 394.81, 366.82, 257.80, 184.10, 156.02, and 283.20. HRMS (ESI^+^): *m*/*z* calcd. for C_23_H_18_IN_4_O_5_ [M+H]^+^: 557.03220; found: 557.03190.

5-nitro-1-((1-(3-((2-oxo-2*H*-chromen-4-yl)oxy)propyl)-1*H*-1,2,3-triazol-4-yl)methyl)indoline-2,3-dione (**6b6**).

Yield: 75%. Light-yellow solid. Melting point: 190.2–193.4 °C. FT-IR (ATR) ν_max (cm^−1^): 775, 1230, 1377, 1458, 1603, 1696, 2925, and 3051. ^1^NMR (400 MHz, DMSO-*d_6_*) δ (ppm): 2.33–2.37 (2H, m, CH_2_), 4.20 (2H, t, *J* = 6.4 Hz, CH_2_), 4.57 (2H, t, *J* = 6.6 Hz, CH_2_), 5.02 (2H, s, CH_2_), 5.82 (1H, s, CH), 7.31–7.37 (3H, m, ArH), 7.63 (1H, t, *J* = 7,2 Hz, ArH), 7.72 (1H, d, *J* = 6.6 Hz, ArH), 8.23 (2H, d, *J* = 7.2 Hz, ArH), 8.49 (1H, dd, *J* = 2.4 Hz, ArH) (Figure S13a). ^13^C NMR (100 MHz, DMSO-*d_6_*) δ (ppm): 29.14, 35.87, 47.15, 67.12, 91.03, 111.97, 115.50, 116.85, 118.43, 119.62, 123.43, 124.51, 133.24, 141.42, 143.58, 153.15, 154.76, 158.72, 161.96, 165.14, 181.31 (Figure S13b). *m*/*z* = 476.1 [M+H]^+^, 313.92, 285.97, 239.97, 183.99, 156.04, and 176.92. HRMS (ESI^+^): *m*/*z* calcd. for C_23_H_18_N_5_O_7_ [M+H]^+^: 476.12063; found: 476.12058.

5-methyl-1-((1-(3-((2-oxo-2*H*-chromen-4-yl)oxy)propyl)-1*H*-1,2,3-triazol-4-yl)methyl)indoline-2,3-dione (**6b7**).

Yield: 89%. Orange solid. Melting point: 207.2–208.0 °C. FT-IR (ATR) ν_max (cm^−1^): 766, 1237, 1489, 1618, 1712, 2923, and 3161. ^1^NMR (400 MHz, DMSO-*d_6_*) δ (ppm): 2.24 (3H, s, Ar–CH_3_), 2.32–2.39 (2H, m CH_2_), 4.18 (2H, t, *J* = 6.4 Hz, CH_2_), 4.56(2H, t, *J* = 6.8 Hz, CH_2_), 4.89 (2H, s, CH_2_), 5.82 (1H, s, CH), 6.99 (1H, d, *J* = 8.4 Hz, ArH), 7.30–7.39 (4H, m, ArH), 7.61–7.65 (1H, m, ArH), 7.69–7.71 (1H, m, ArH), 8.21 (1H, s, ArH) (Figure S14a). ^13^C NMR (100 MHz, DMSO-*d_6_*) δ (ppm): 20.48, 29.11, 35.47, 47.14, 67.15, 91.02, 111.41, 115.51, 116.84, 117.92, 123.44, 124.32, 124.51, 125.13, 133.23, 138.75, 141.90, 148.45, 153.16, 158.22, 162.00, 165.17, 183.74 (Figure S14b). *m*/*z* = 445.1 [M+H]^+^, 282.99, 255.01, 172.07, 146.11, 199.04, and 171.04. HRMS (ESI^+^): *m*/*z* calcd. for C_24_H_21_N_4_O_5_ [M+H]^+^: 445.15120; found: 445.15110.

1-((1-(4-((2-oxo-2*H*-chromen-4-yl)oxy)butyl)-1*H*-1,2,3-triazol-4-yl)methyl)indoline-2,3-dione (**6c1**).

Yield: 93%. Light-orange solid. Melting point: 198.8–199.6 °C. FT-IR (ATR) ν_max (cm^−1^): 761, 928, 1187, 1465, 1608, 1708, 2950, and 3093. ^1^NMR (400 MHz, DMSO-*d_6_*) δ (ppm): 1.74–1.79 (2H, m, CH_2_), 1.95–2.03 (2H, m, CH_2_), 4.19–4.22 (2H, t, *J* = 6.4 Hz, CH_2_), 4.40–4.44 (2H, t, *J* = 6.8 Hz, CH_2_), 4.95 (2H, s, CH_2_), 5.86 (1H, s, CH), 7.08–7.16 (2H, m, ArH), 7.33–7.40 (2H, m, ArH), 7.54–7.67 (3H, m, ArH), 7.76–7.79 (1H, m, ArH), 8.19 (1H, s, ArH) (Figure S15a). ^13^C NMR (100 MHz, DMSO-*d_6_*) δ (ppm): 25.44, 26.73, 35.54, 49.46, 69.19, 91.01, 111.61, 115.65, 116.90, 118.04, 123.30, 123.80, 124.02, 124.65, 124.90, 133.20, 138.50, 141.88, 150.64, 153.20, 158.23, 162.09, 165.31, 183.55 (Figure S15b). *m*/*z* = 445.1 [M+H]^+^, 283.03, 255.06, 157.12, 185.05, 132.15, and 199.10. HRMS (ESI^+^): *m*/*z* calcd. for C_24_H_21_N_4_O_5_ [M+H]^+^: 445.15120; found: 445.15115.

5-fluoro-1-((1-(4-((2-oxo-2*H*-chromen-4-yl)oxy)butyl)-1*H*-1,2,3-triazol-4-yl)methyl)indoline-2,3-dione (**6c2**).

Yield: 78%. Orange solid. Melting point: 210.1–212.4 °C. FT-IR (ATR) ν_max (cm^−1^): 766, 924, 1265, 1485, 1621, 1707, 2950, and 3087. ^1^NMR (400 MHz, DMSO-*d_6_*) δ (ppm): 1.72–1.79 (2H, m, CH_2_), 1.95–2.02 (2H, m, CH_2_), 4.19–4.22 (2H, t, *J* = 6.4 Hz, CH_2_), 4.40–4.44 (2H, t, *J* = 6.8 Hz, CH_2_), 4.95 (2H, s, CH_2_), 5.86 (1H, s, CH), 7.15–7.18 (1H, m, ArH), 7.32–7.39 (2H, m, ArH), 7.44–7.51 (2H, m, ArH), 7.62–7.66 (1H, m, ArH), 7.78 (1H, d, *J* = 6.8 Hz, ArH), 8.19 (1H, s, ArH) (Figure S16a). ^13^C NMR (100 MHz, DMSO-*d_6_*) δ (ppm): 25.44, 26.73, 35.65, 49.47, 69.18, 91.02, 113.33, 115.66, 116.91, 119.47, 123.30, 124.04, 124.40, 124.65, 128.04, 133.20, 137.32, 141.71, 149.13, 153.22, 158.02, 162.09, 165.31, 182.44 (Figure S16b). *m*/*z* = 463.1 [M+H]^+^, 301.02, 273.04, 175.09, 203.01, 173.08, and 150.09. HRMS (ESI^+^): *m*/*z* calcd. for C_24_H_20_FN_4_O_5_ [M+H]^+^: 463.14177; found: 463.14183.

5-chloro-1-((1-(4-((2-oxo-2*H*-chromen-4-yl)oxy)butyl)-1*H*-1,2,3-triazol-4-yl)methyl)indoline-2,3-dione (**6c3**).

Yield: 77%. Orange solid. Melting point: 210.9–212.7 °C. FT-IR (ATR) ν_max (cm^−1^): 770, 934, 1247, 1437, 1605, 1728, 2945, and 3075. ^1^NMR (400 MHz, DMSO-*d_6_*) δ (ppm): 1.72–1.79 (2H, m, CH_2_), 1.95–2.02 (2H, m, CH_2_), 4.18–4.21 (2H, t, *J* = 6.4 Hz, CH_2_), 4.40–4.43 (2H, t, *J* = 6.8 Hz, CH_2_), 4.96 (2H, s, CH_2_), 5.85 (1H, s, CH), 7.18 (1H, d, *J* = 8.4 Hz, ArH), 7.32–7.39 (2H, m, ArH), 7.60 (1H, d, *J* = 2.0 Hz, ArH), 7.62–7.67 (1H, m, ArH), 7.75–7.78 (2H, m, ArH), 8.19 (1H, s, ArH) (Figure S17a). ^13^C NMR (100 MHz, DMSO-*d_6_*) δ (ppm): 25.44, 26.73, 35.65, 49.47, 69.18, 91.02, 113.33, 115.66, 116.91, 119.47, 123.30, 124.04, 124.40, 124.65, 128.04, 133.20, 137.32, 141.71, 149.13, 158.02, 162.09, 165.32, 182.44 (Figure S17b). *m*/*z* = 479.1 [M+H]^+^, 316.96, 288.99, 191.02, 189.03, 218.99, and 166.04. HRMS (ESI^+^): *m*/*z* calcd. for C_24_H_20_ClN_4_O_5_ [M+H]^+^: 479.11222; found: 479.11228.

5-bromo-1-((1-(4-((2-oxo-2*H*-chromen-4-yl)oxy)butyl)-1*H*-1,2,3-triazol-4-yl)methyl)indoline-2,3-dione (**6c4**).

Yield: 81%. Red solid. Melting point: 207.8–209.2 °C. FT-IR (ATR) ν_max (cm^−1^): 769, 934, 1247, 1444, 1604, 1727, 2942, and 3074. ^1^NMR (400 MHz, DMSO-*d_6_*) δ (ppm): 1.74–1.79 (2H, m, CH_2_), 1.95–2.02 (2H, m, CH_2_), 4.20 (2H, t, *J* = 6.4 Hz, CH_2_), 4.42 (2H, t, *J* = 6.8 Hz, CH_2_), 4.95 (2H, s, CH_2_), 5.86 (1H, s, CH), 7.13 (1H, d, *J* = 8.4 Hz, ArH), 7.33–7.40 (2H, m, ArH), 7.63–7.70 (2H, m, ArH), 7.76–7.80 (2H, m, ArH), 8.19 (1H, s, ArH) (Figure S18a). ^13^C NMR (100 MHz, DMSO-*d_6_*) δ (ppm): 25.43, 26.73, 35.62, 49.46, 69.17, 91.02, 113.75, 115.56, 115.65, 116.91, 119.85, 123.30, 124.04, 124.65, 127.13, 133.21, 140.13, 141.74, 149.49, 153.21, 157.85, 162.09, 165.31, 182.29 (Figure S18b). *m*/*z* = 523.0 [M+H]^+^, 360.89, 332.91, 209.88, 232.91, 262.87, and 234.77. HRMS (ESI^+^): *m*/*z* calcd. for C_24_H_20_BrN_4_O_5_ [M+H]^+^: 523.06171; found: 523.06156.

5-iodo-1-((1-(4-((2-oxo-2*H*-chromen-4-yl)oxy)butyl)-1*H*-1,2,3-triazol-4-yl)methyl)indoline-2,3-dione (**6c5**).

Yield: 72%. Orange solid. Melting point: 235.1–238.1 °C. FT-IR (ATR) ν_max (cm^−1^): 751, 928, 1248, 1462, 1601, 1713, 2921, and 3145. ^1^NMR (400 MHz, DMSO-*d_6_*) δ (ppm): 1.74–1.79 (2H, m, CH_2_), 1.95–2.00 (2H, m, CH_2_), 4.20 (2H, t, *J* = 6.0 Hz, CH_2_), 4.42 (2H, t, *J* = 7.0 Hz, CH_2_), 4.94 (2H, s, CH_2_), 5.86 (1H, s, CH), 7.00 (1H, d, *J* = 8.4 Hz, ArH), 7.33–7.40 (2H, m, ArH), 7.65 (1H, t, *J* = 7.8, ArH), 7.78 (2H, t, *J* = 7.0 Hz ArH), 7.94 (1H, d, *J* = 8.4 Hz, ArH), 8.18 (1H, s, ArH) (Figure S19a). ^13^C NMR (100 MHz, DMSO-*d_6_*) δ (ppm): 25.44, 26.73, 35.59, 49.47, 69.18, 86.76, 91.03, 114.07, 115.67, 116.91, 120.11, 123.31, 124.00, 124.65, 132.58, 133.20, 141.71, 145.92, 149.92, 153.22, 157.57, 162.08, 165.32, 182.20 (Figure S19b). *m*/*z* = 571.0 [M+H]^+^, 408.86, 380.86, 280.86, 257.77, 198.04, and 197.01. HRMS (ESI^+^): *m*/*z* calcd. for C_24_H_20_IN_4_O_5_ [M+H]^+^: 571.04785; found: 571.04766.

5-nitro-1-((1-(4-((2-oxo-2*H*-chromen-4-yl)oxy)butyl)-1*H*-1,2,3-triazol-4-yl)methyl)indoline-2,3-dione (**6c6**).

Yield: 72%. Light-yellow solid. Melting point: 218.3–220.2 °C. FT-IR (ATR) ν_max (cm^−1^): 772, 928, 1234, 1330, 1604, 1702, 2918, and 3103. ^1^NMR (400 MHz, DMSO-*d_6_*) δ (ppm): 1.73–1.79 (2H, m, CH_2_), 1.95–2.02 (2H, m, CH_2_), 4.20 (2H, t, *J* = 6.2 Hz, CH_2_), 4.43 (2H, t, *J* = 6.8 Hz, CH_2_), 5.05 (2H, s, CH_2_), 5.85 (1H, s, CH), 7.32–7.39 (3H, m, ArH), 7.64 (1H, t, *J* = 7.6 Hz, ArH), 8.22 (2H, t, *J* = 8.0 Hz, ArH), 8.48 (1H, t, *J* = 7.6 Hz, ArH), 8.50 (1H, s, ArH) (Figure S20a). ^13^C NMR (100 MHz, DMSO-*d_6_*) δ (ppm): 25.44, 26.74, 35.94, 49.49, 69.17, 91.01, 112.03, 115.64, 116.91, 118.52, 119.60, 123.27, 124.15, 124.62, 133.20, 133.27, 141.45, 143.56, 153.20, 154.82, 158.80, 162.07, 165.29, 181.33 (Figure S20b). *m*/*z* = 490.3 [M+H]^+^, 286.44, 455.29, 298.90, 283.15, 290.71, and 374.65. HRMS (ESI^+^): *m*/*z* calcd. for C_24_H_20_N_5_O_7_ [M+H]^+^: 490.13628; found: 490.13625.

5-methyl-1-((1-(4-((2-oxo-2*H*-chromen-4-yl)oxy)butyl)-1*H*-1,2,3-triazol-4-yl)methyl)indoline-2,3-dione (**6c7**).

Yield: 85%. Orange solid. Melting point: 225.0–227.7 °C. FT-IR (ATR) ν_max (cm^−1^): 764, 915, 1248, 1489, 1617, 1713, 2922, and 3072. ^1^NMR (400 MHz, DMSO-*d_6_*) δ (ppm): 1.74–1.79 (2H, m, CH_2_), 1.95–2.02 (2H, m, CH_2_), 2.23 (3H, s, Ar-CH_3_), 4.20 (2H, t, *J* = 6.2 Hz, CH_2_), 4.41 (2H, t, *J* = 7.0 Hz, CH_2_), 4.93 (2H, s, CH_2_), 5.86 (1H, s, CH), 7.04 (1H, d, *J* = 8.4 Hz, ArH), 7.33–7.42 (4H, m, ArH), 7.63–7.76 (1H, m, ArH), 7.78 (1H, d, *J* = 6.4 Hz, ArH), 8.19 (1H, s, ArH) (Figure S21a). ^13^C NMR (100 MHz, DMSO-*d_6_*) δ (ppm): 20.46, 25.44, 26.72, 35.54, 49.45, 69.18, 91.01, 111.48, 115.66, 116.90, 117.99, 123.31, 123.98, 124.65, 125.11, 133.20, 138.78, 141.93, 148.51, 153.21, 158.28, 162.08, 165.32, 183.78 (Figure S21b). *m*/*z* = 459.1 [M+H]^+^, 297.02, 269.06, 199.04, 171.09, 213.10, and 169.14. HRMS (ESI^+^): *m*/*z* calcd. for C_25_H_23_N_4_O_5_ [M+H]^+^: 459.16685; found: 459.16688.

### 3.3. Biological Assays

#### 3.3.1. Enzymatic Assays

- In Vitro Inhibitory Activity Against AChE and BChE

The in vitro cholinesterase inhibitory activity of the synthesized hybrid molecules was evaluated using a modified Ellman’s assay, optimized for 96-well microplate application [64]. AChE from *Electrophorus electricus* (AChE, EC 3.1.1.7), BChE from equine serum (BChE, EC 3.1.1.8), acetylthiocholine iodide (ATCh), butyrylthiocholine iodide (BTCh), and 5,5′-dithiobis(2-nitrobenzoic acid) were purchased from Sigma-Aldrich (St. Louis, MO, USA) and used without further purification.

Test compounds were initially dissolved in dimethylsulphoxide (DMSO) to yield 1 mM stock solutions and subsequently diluted with 0.1 M phosphate buffer (pH 8.0) to prepare working solutions. Acetylthiocholine iodide (ATCh15 mM) and butyrylthiocholine iodide (BTCh, 15 mM) were dissolved in deionized water, while DTNB (10 mM) and enzyme stock solutions (100 U/L) were freshly prepared in 0.1 M phosphate buffer (pH 8.0).

For the assay, 20 µL of compound solution was added to 140 µL of 0.1 M phosphate buffer (pH 8.0) in each well, followed by 20 µL of enzyme solution (AChE or BuChE, 100 U/L). After incubation at room temperature for 10 min, the reaction was initiated by adding 10 µL of DTNB (0.01 M) and 10 µL of substrate (ATCh or BTCh, 0.015 M). The formation of the 5-thio-2-nitrobenzoate anion, resulting from the enzymatic hydrolysis of the substrate and subsequent reaction with DTNB, was monitored spectrophotometrically at 405 nm for 20 min using a VICTOR^®^ multimode plate reader (PerkinElmer, Shelton, CT, USA). The percentage inhibition of enzyme activity was calculated using the following equation:

(1) $$\%\;Inhibition=\left(\frac{Absorbance\;of\;control-Apsorbance\;of\;sample}{Absorbance\;of\;control}\right)\times 100$$

where the absorbance of the control represents the maximum enzyme activity (without inhibition), and the absorbance of the sample corresponds to the enzyme activity in the presence of the tested compound. Appropriate blanks were included to account for background absorbance and to correct for any intrinsic color of the tested compounds.

Compounds exhibiting more than 50% inhibition at 20 µM were selected for further analysis to determine their IC_50_ (the concentration of inhibitor needed to reduce enzyme activity by half). Additional dilutions were tested to achieve at least 20–80% inhibition, and IC_50_ values were determined using a nonlinear regression method by plotting enzyme inhibition (%) against compound concentration (log transformed) using GraphPad 10.4.2 (GraphPad Software, San Diego, CA, USA). All experiments were performed in triplicate.

- Type of Enzyme Inhibition

The type of enzyme inhibition was assessed using Lineweaver–Burk plots, which represent a linear transformation of the Michaelis–Menten equation. This method enables visual classification of inhibition type by plotting the reciprocal of the initial reaction velocity (1/*Vo*) against the reciprocal of the substrate concentration (1/[S]).

The study was conducted for the most active compound against BChE (**6c1**), using substrate concentrations of 1 mM, 2 mM, 4 mM, and 8 mM, in the absence and presence of the inhibitor at 4 μM, 8 μM, and 12 μM. The intersection pattern of the fitted lines was used to identify the inhibition mechanism. All tests were replicated three times.

#### 3.3.2. Antimicrobial Screening

The antimicrobial potential of the synthesized coumarin–triazole–isatin hybrids was evaluated by the agar well diffusion method. The activity was tested against a variety of Gram-positive bacteria (*Staphylococcus aureus* ATCC 25923 and ATCC 902710, *Staphylococcus epidermidis* ATCC 12228, *Streptococcus pyogenes* ATCC 19615, *Enterococcus faecalis* ATCC 51299), Gram-negative bacteria (*Escherichia coli* ATCC 25922, *Haemophilus influenzae* ATCC 49766, *Pseudomonas aeruginosa* ATCC 27853, *Salmonella typhi* ATCC 14028, *Neisseria meningitidis* ATCC 13090, *Neisseria gonorrhoeae* ATCC 43069), and fungi (*Candida albicans* ATCC 60193 and ATCC 90028, *Candida orientalis* ATCC 6258). All microbial strains were obtained from Microbiologics (St.Cloud, MN, USA).

The test compounds were dissolved in DMSO to obtain stock solutions (1 mg/mL). Each compound (60 µL) was transferred into wells (6 mm in diameter) that were previously perforated into agar plates inoculated with microbial suspensions standardized to 0.5 McFarland for bacteria and 2 McFarland for *Candida* spp. DMSO was used as a negative control, while commercial antibiotic disks of novobiocin (5 μg), ampicillin (10 μg), and ciprofloxacin (5 μg) served as positive controls. Antimicrobial activity was assessed by measuring the diameter of zones of inhibition (ZOIs) in millimeters. All tests were conducted in triplicate.

The agar dilution method was performed only for the compounds that exhibited the most pronounced antimicrobial activity in the agar diffusion assay, and only against those bacterial strains for which satisfactory ZOIs were observed. Mueller–Hinton agar plates were prepared with test compounds in a concentration range of 0.39–200 μg/mL, inoculated with 5 × 10^4^ CFU/mL microbial suspensions, and incubated under the same conditions; the minimum inhibitory concentration (MIC) was defined as the lowest concentration at which no visible microbial growth was observed.

### 3.4. Molecular Docking

Molecular docking analyses were performed to examine the binding interactions of compound **6c1** with AChE and BChE using high-resolution crystal structures retrieved from the Protein Data Bank (PDB) [76]: 5NAP (*Tc*AChE complexed with a non-chiral donepezil-like inhibitor 17, resolution 2.17 Å) [77] and 5K5E (*h*BChE bound to 6QS, resolution 2.8 Å) [78]. Enzyme structures were refined and prepared for docking simulations using MAKE Receptor 3.2.0.2 software [79]. Water molecules were removed, and hydrogen atoms were added according to standard protonation states at physiological pH. Molecular docking analyses were conducted within grid boxes centered around the co-crystallized ligands, with dimensions of 20.90 Å × 14.29 Å × 20.68 Å for AChE and 17.85 Å × 20.17 Å × 16.33 Å for BChE. The outer contour volumes were calculated as 837 Å^3^ and 733 Å^3^, respectively. Docking site shape parameters were set to a balanced configuration, with no additional constraints applied to ensure flexibility in ligand accommodation.

Molecular docking studies were conducted using OpenEye’s OEDocking 3.2.0.2 software [80,81,82]. Ligand conformations were pre-generated with OMEGA 2.5.1.4 [83,84], and docking was performed using the fast rigid exhaustive docking (FRED) tool. The docking poses were scored using the ChemGauss4 scoring function (Table S1) [72].

To validate the docking protocol, the native conformations of the non-chiral donepezil-like inhibitor 17 and 6QS were re-docked into the active sites of AChE and BChE, respectively. The docking-generated conformers of these co-crystallized ligands were then aligned with their corresponding experimental crystal structures, and root-mean-square deviation (RMSD) values were calculated. The obtained RMSD values were below 2.0 Å, confirming the reliability of the docking approach used in this study [85].

## 4. Conclusions

In this study, a novel series of 21 hybrid molecules was synthesized, structurally characterized, and evaluated for its potential as multifunctional agents in the treatment of AD. The design rationale was based on combining three pharmacophores—coumarin, triazole, and isatin—privileged scaffolds in medicinal chemistry known for their broad biological activity, including cholinesterase inhibition. Structural modifications included systematic variation in the linker length and substituent at the position 5 of the isatin ring, allowing detailed investigation of SAR. Biological screening revealed that several compounds demonstrated pronounced inhibition of BChE with selectivity over AChE. Notably, compound **6c1** emerged as the most active BChE inhibitor (IC_50_ = 1.74 μM), showing superior potency than the reference drug donepezil. In contrast, the compounds displayed weaker AChE inhibition, underscoring the scaffold’s preferential affinity for BChE.

SAR analysis highlighted the importance of linker length and steric/electronic characteristics of the isatin substituents. Longer linkers enhanced inhibitory potency, likely due to improved conformational adaptability within the enzyme active site. Unsubstituted and small halogenated derivatives generally showed superior activity, whereas bulky or strongly electron-withdrawing groups negatively impacted binding affinity. Enzyme kinetics confirmed that the most potent compounds acted as mixed-type reversible inhibitor, and molecular docking supported dual-site binding within BChE. Involvement of crucial interactions with residues from CAS and PAS further explained the observed bioactivity trends.

Given their novel structures, favorable in vitro potency, and BChE selectivity, these compounds—particularly **6c1**—represent promising leads for the development of selective BChE inhibitors. Further studies including pharmacokinetic profiling, blood–brain barrier permeability assessment, and in vivo efficacy models will be crucial to validate their therapeutic potential as multi-target agents for the symptomatic and disease-modifying treatment of AD.

## 5. Patent

National Patent Application: MK/P/2024/000380.

## Figures and Tables

**Table 1:** AChE and BChE inhibitory activity of the compounds **6a1**–**6c7**.

Compound	BChE		AChE		Selectivity BChE/AChE
	% of Inhibition (Conc. 20 μM)	IC_50_ μM	% of Inhibition (Conc. 20 μM)	IC_50_ μM	
**6a1**	72.81 ± 2.17	6.08 ± 1.19	29.27 ± 2.14	-	-
**6a2**	66.15 ± 5.18	9.16 ± 1.69	11.78 ± 1.18	-	-
**6a3**	62.65 ± 6.19	10.58 ± 1.13	5.57 ± 1.19	-	-
**6a4**	22.70 ± 3.87	-	4.57 ± 0.87	-	-
**6a5**	40.89 ± 6.26	-	3.17 ± 0.26	-	-
**6a6**	4.89 ± 0.74	-	3.11 ± 0.44	-	-
**6a7**	2.91 ± 0.15	-	22.76 ± 3.66	-	-
**6b1**	69.23 ± 5.27	8.52 ± 1.28	69.23 ± 4.27	18.16 ± 1.19	2.13
**6b2**	75.51 ± 5.69	5.90 ± 1.09	44.27 ± 5.69	-	-
**6b3**	61.64 ± 7.24	11.25 ± 1.16	31.50 ± 3.24	-	-
**6b4**	66.22 ± 8.11	13.28 ± 2.19	22.91 ± 2.11	-	-
**6b5**	61.65 ± 7.49	14.84 ± 1.44	27.58 ± 1.49	-	-
**6b6**	5.89 ± 1.91	-	17.88 ± 1.91	-	-
**6b7**	4.88 ± 0.27	-	24.41 ± 1.27	-	-
**6c1**	84.52 ± 8.46	1.74 ± 0.29	67.12 ± 3.27	15.28 ± 1.22	8.78
**6c2**	79.13 ± 5.49	5.51 ± 1.38	60.98 ± 3.49	17.13 ± 1.65	3.11
**6c3**	5.19 ± 1.14	-	44.23 ± 3.14	-	-
**6c4**	27.10 ± 2.19	-	9.84 ± 1.19	-	-
**6c5**	22.93 ± 1.64	-	11.21 ± 1.64	-	-
**6c6**	4.15 ± 0.54	-	18.98 ± 1.96	-	-
**6c7**	10.32 ± 1.53	-	12.12 ± 1.26	-	-
Donepezil	74.22 ± 6.17	5.24 ± 1.16	~100	0.08 ± 0.02	-

All values are presented as average ± standard error (*n* = 3).

**Table 2:** Antibacterial activity of the compounds **6a1**–**6c7**.

Compound	*Staphylococcus aureus* ATCC 25923 ZOI (mm ± SD)	*Staphylococcus epidermidis* ATCC 12228 ZOI (mm ± SD)
**6a1**	*	6.08 ± 1.19
**6a2**	*	9.16 ± 1.69
**6a3**	*	10.58 ± 1.13
**6a4**	12.67 ± 2.31	*
**6a5**	*	*
**6a6**	*	*
**6a7**	*	*
**6b1**	*	8.52 ± 1.28
**6b2**	9.33 ± 4.04	5.90 ± 1.09
**6b3**	10.33 ± 1.15	11.25 ± 1.16
**6b4**	20.00 ± 3.61	13.28 ± 2.19
**6b5**	*	14.84 ± 1.44
**6b6**	*	*
**6b7**	*	*
**6c1**	8.67 ± 1.15	1.74 ± 0.29
**6c2**	12.00 ± 2.65	5.51 ± 1.38
**6c3**	10.67 ± 3.06	*
**6c4**	12.67 ± 0.58	*
**6c5**	*	*
**6c6**	*	*
**6c7**	*	*
Ciprofloxacine	27.00 ± 1.62	5.24 ± 1.16

All values are presented as average ± standard deviation (*n* = 3). ZOI was measured including the well (6 mm). * No ZOI was observed

## Data Availability

The original contributions presented in this study are included in the article/Supplementary Material. Further inquiries can be directed to the corresponding author.

## Supplementary Materials

The following supporting information can be downloaded at https://www.mdpi.com/article/10.3390/molecules30102121/s1, Schemes S1–S4: Synthetic schemes, Figures S1–S21: ^1^H NMR spectra (a) and ^13^C NMR (b) of compounds 6a1–6c7, Figures S22 and S23: Dose–inhibition curves for the determination of IC_50_ values, Figures S24 and S25: 2D interaction diagrams of inactive compounds 6c4, 6c5, 6c6 and 6c7 docked into hBChE, PDB ID: 5K5E and interaction diagrams of inactive compounds 6a3, 6a4, 6a5, 6a6 and 6a7 docked into *Tc*AChE, PDB ID: 5NAP and Table S1: ChemGauss4 Scores for Docking to hBChE and *Tc*AChE.
