# Supplementary material for: Synthesis, Biological Evaluation, and Molecular Docking Studies of Novel Coumarin–Triazole–Isatin Hybrids as Selective Butyrylcholinesterase Inhibitors
Source: Molecules. 2025 May 11;30(10):2121. doi: 10.3390/molecules30102121 (PMC12114297; doi:10.3390/molecules30102121)
Supplement: Supplementary file 1 [file molecules-30-02121-s001.zip › molecules-3622449-supplementary.pdf]

MOLECULES

Supplementary Information associated with the paper

Synthesis, Biological Evaluation and Molecular Docking Studies of Novel Coumarin–Triazole–Isatin Hybrids as Selective Butyrylcholinesterase Inhibitors

Aleksandar Dimkovski [1], Vladimir Dobričić [2], Milena Simić [3], Maja Jurhar Pavlova [4], Evgenija Mihajloska [1], Zoran Sterjev [1], Ana Poceva Panovska [5]

[1] Ss Cyril and Methodius University in Skopje, Faculty of Pharmacy, Institute for Pharmaceutical Chemistry, Majka Tereza 47, 1000, Skopje, Republic of North Macedonia.

[2] University of Belgrade, Faculty of Pharmacy, Department of Pharmaceutical Chemistry, Vojvode Stepe 450, 11221, Belgrade, Serbia.

[3] University of Belgrade, Faculty of Pharmacy, Department of Organic Chemistry, Vojvode Stepe 450, 11221, Belgrade, Serbia.

[4] Ss Cyril and Methodius University in Skopje, Faculty of Medicine, Institute for Microbiology and Parasitology, 50 Divizija 6, 1000, Skopje, Republic of North Macedonia.

[5] Ss Cyril and Methodius University in Skopje, Faculty of Pharmacy, Institute for Applied Chemistry and Pharmaceutical Analysis, Majka Tereza 47, 1000, Skopje, Republic of North Macedonia.

*Correspondence: aleksandar.d@ff.ukim.edu.mk

Contents

Synthetic schemes

Synthetic scheme for compounds **2a-2g** 4
Synthetic scheme for compounds **4a-4c** 4
Synthetic scheme for compounds **5a-5c** 4
Synthetic scheme for compounds **6a1-6c7** 5

NMR spectra of compounds 6a1-6c7

^{1}H NMR spectrum (a) and ^{13}C NMR (b) of compound **6a1** 6
^{1}H NMR spectrum (a) and ^{13}C NMR (b) of compound **6a2** 7
^{1}H NMR spectrum (a) and ^{13}C NMR (b) of compound **6a3** 8
^{1}H NMR spectrum (a) and ^{13}C NMR (b) of compound **6a3** 9
^{1}H NMR spectrum (a) and ^{13}C NMR (b) of compound **6a5** 10
^{1}H NMR spectrum (a) and ^{13}C NMR (b) of compound **6a6** 11
^{1}H NMR spectrum (a) and ^{13}C NMR (b) of compound **6a7** 12
^{1}H NMR spectrum (a) and ^{13}C NMR (b) of compound **6b1** 13
^{1}H NMR spectrum (a) and ^{13}C NMR (b) of compound **6b2** 14
^{1}H NMR spectrum (a) and ^{13}C NMR (b) of compound **6b3** 15
^{1}H NMR spectrum (a) and ^{13}C NMR (b) of compound **6b4** 16
^{1}H NMR spectrum (a) and ^{13}C NMR (b) of compound **6b5** 17
^{1}H NMR spectrum (a) and ^{13}C NMR (b) of compound **6b6** 18
^{1}H NMR spectrum (a) and ^{13}C NMR (b) of compound **6b7** 19
^{1}H NMR spectrum (a) and ^{13}C NMR (b) of compound **6c1** 20
^{1}H NMR spectrum (a) and ^{13}C NMR (b) of compound **6c2** 21
^{1}H NMR spectrum (a) and ^{13}C NMR (b) of compound **6c3** 22
^{1}H NMR spectrum (a) and ^{13}C NMR (b) of compound **6c4** 23
^{1}H NMR spectrum (a) and ^{13}C NMR (b) of compound **6c5** 24
^{1}H NMR spectrum (a) and ^{13}C NMR (b) of compound **6c6** 25
^{1}H NMR spectrum (a) and ^{13}C NMR (b) of compound **6c7** 26

Dose-inhibition curves

Dose–inhibition curves for the determination of IC_{50} values of compounds **6a1, 6a2, 6b1, 6b2, 6b3, 6b4, 6b5, 6c1,** and **6c2** against butyrylcholinesterase (BChE) 27
Dose–inhibition curves for the determination of IC_{50} values of compounds **6a1, 6c1,** and **6c2** against acetylcholinesterase (AChE) 29

2D interaction diagrams of inactive compounds docked into active sites of cholinesterases

2D interaction diagrams of inactive compounds **6c4, 6c5, 6c6** and **6c7** docked into the active site of human butyrylcholinesterase (PDB ID: 5K5E) 30
2D interaction diagrams of inactive compounds **6a3, 6a4, 6a5, 6a6** and 6a7 docked into the active site of *Torpedo californica* acetylcholinesterase (TcAChE, PDB ID: 5NAP). 31

ChemGauss4 Scores for Docking to hBChE and TcAChE 33

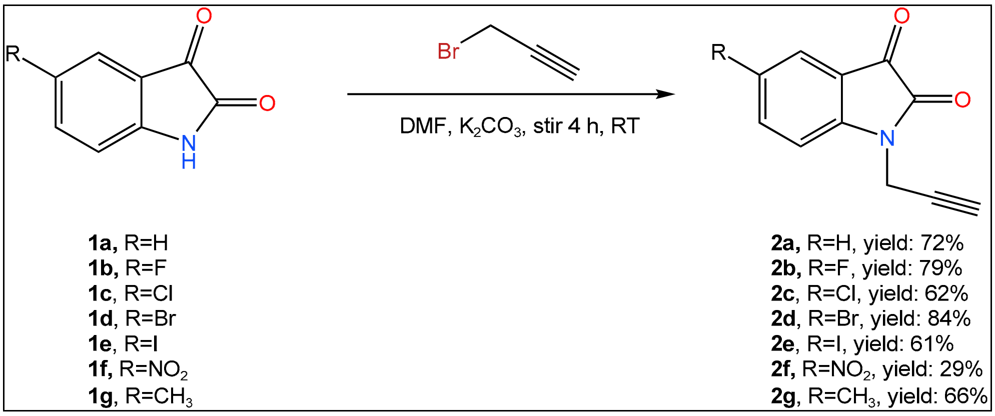

Scheme S1. Synthesis of compounds **2a-2g**

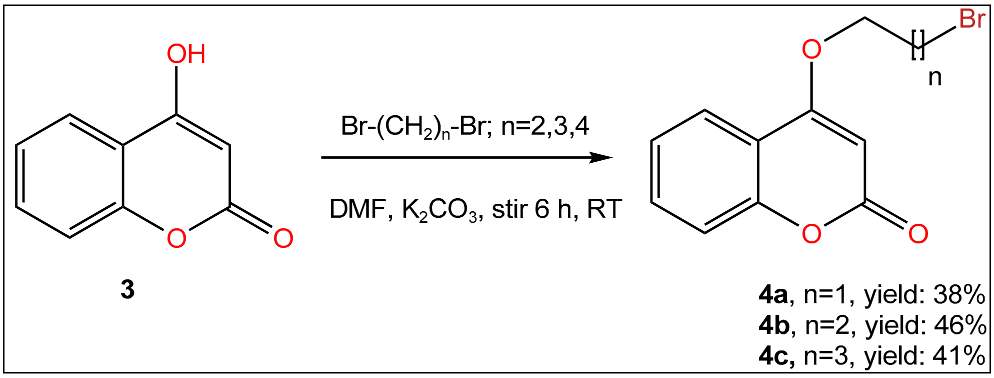

Scheme S2. Synthesis of compounds **4a-4c**

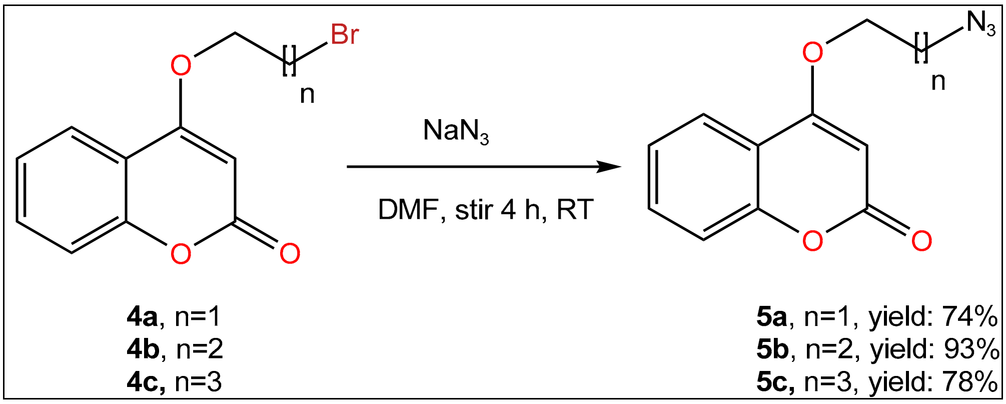

Scheme S3. Synthesis of compounds **5a-5c**

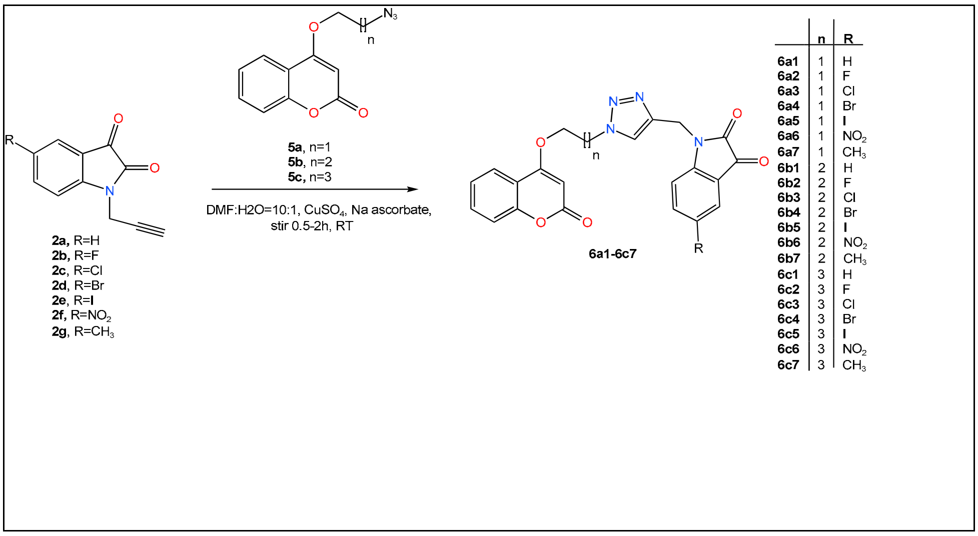

Scheme S4. Synthesis of compounds **6a1-6c7**

a)

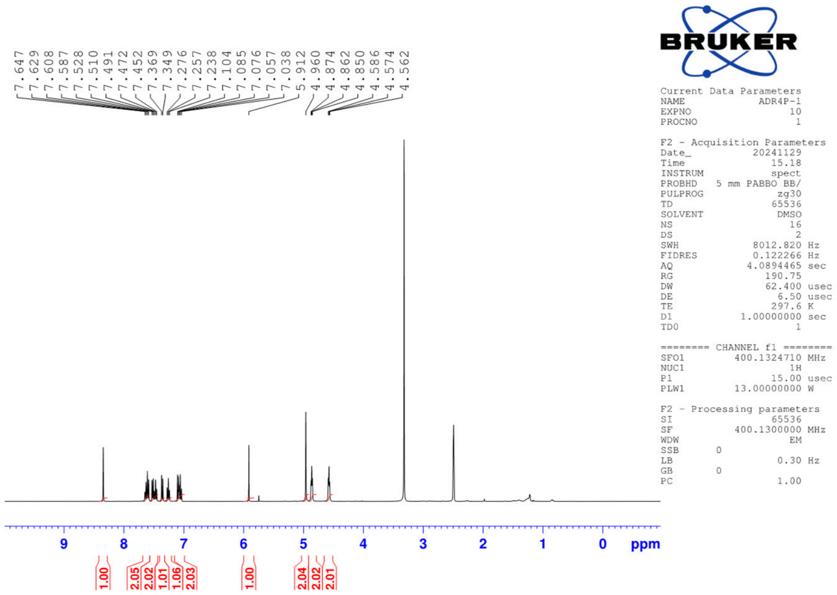

b)

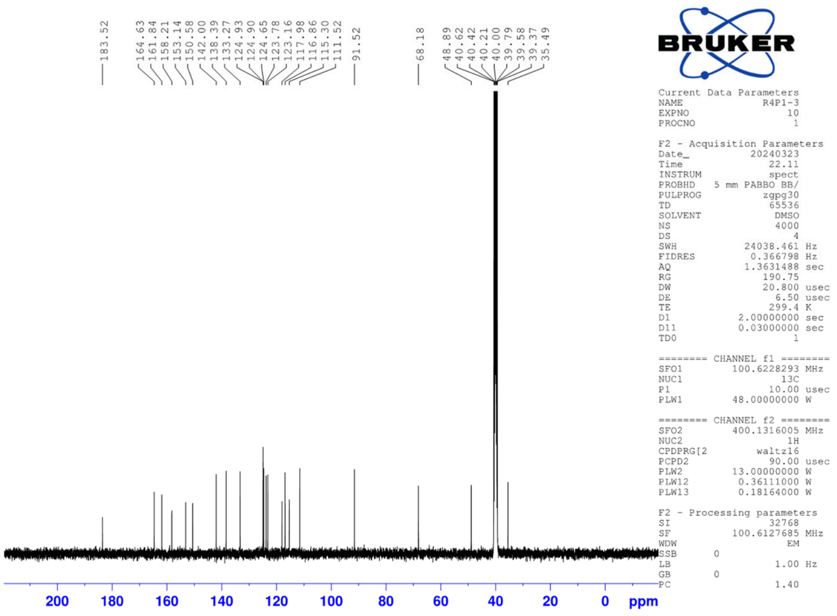

Figure S1. ^{1}H NMR spectrum (a) and ^{13}C NMR (b) of compound **6a1**

a)

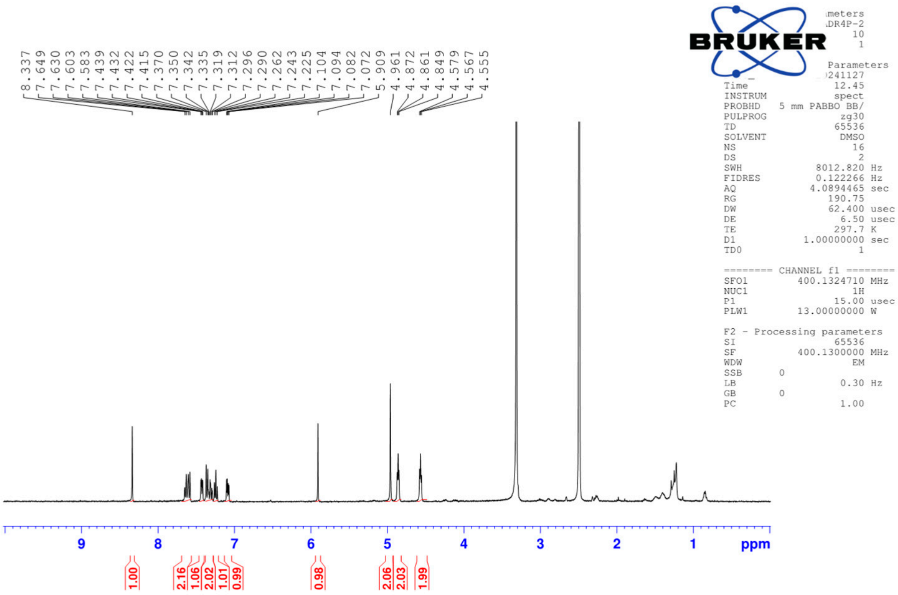

b)

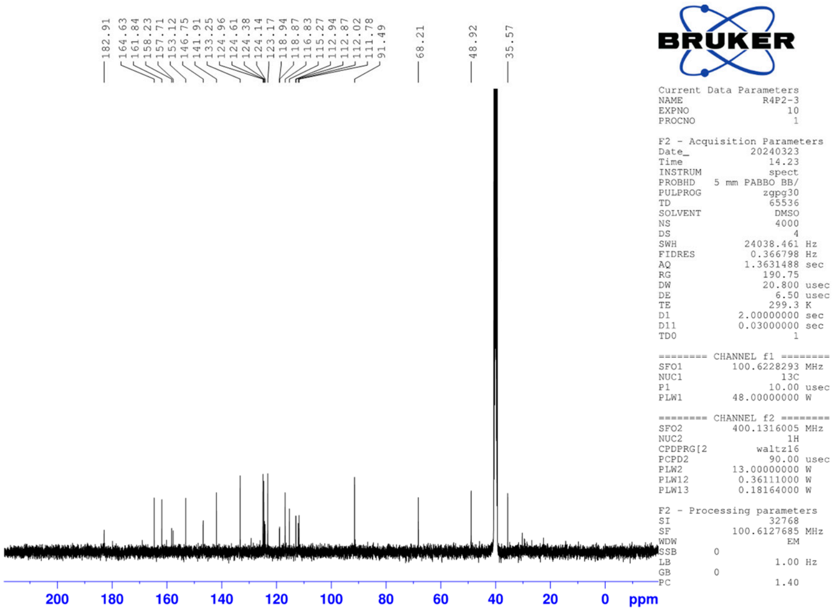

Figure S2. ^{1}H NMR spectrum (a) and ^{13}C NMR (b) of compound **6a2**

a)

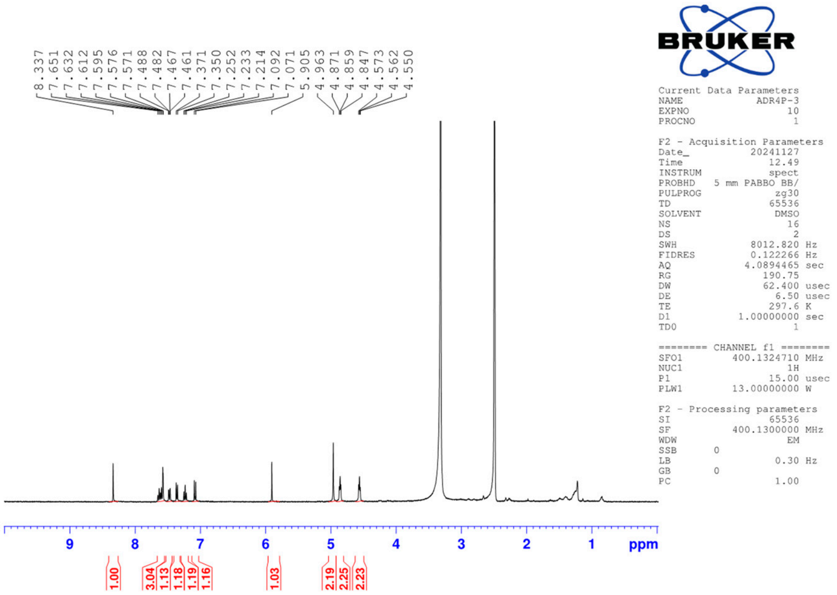

b)

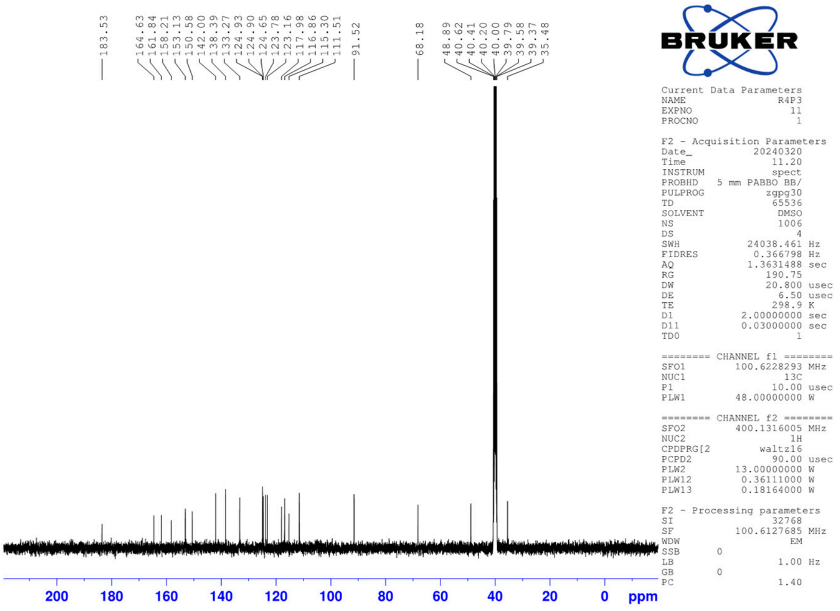

Figure S3. ^{1}H NMR spectrum (a) and ^{13}C NMR (b) of compound **6a3**

a)

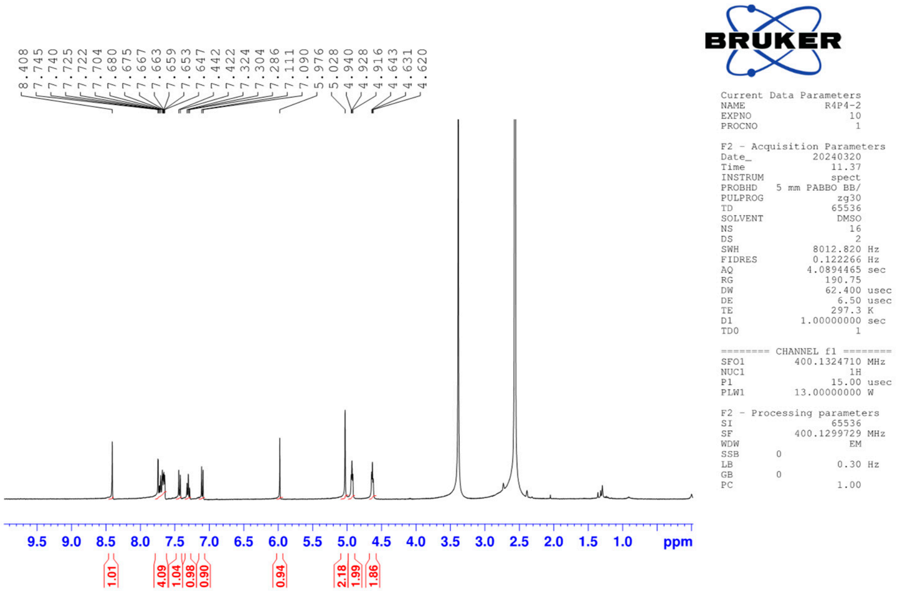

b)

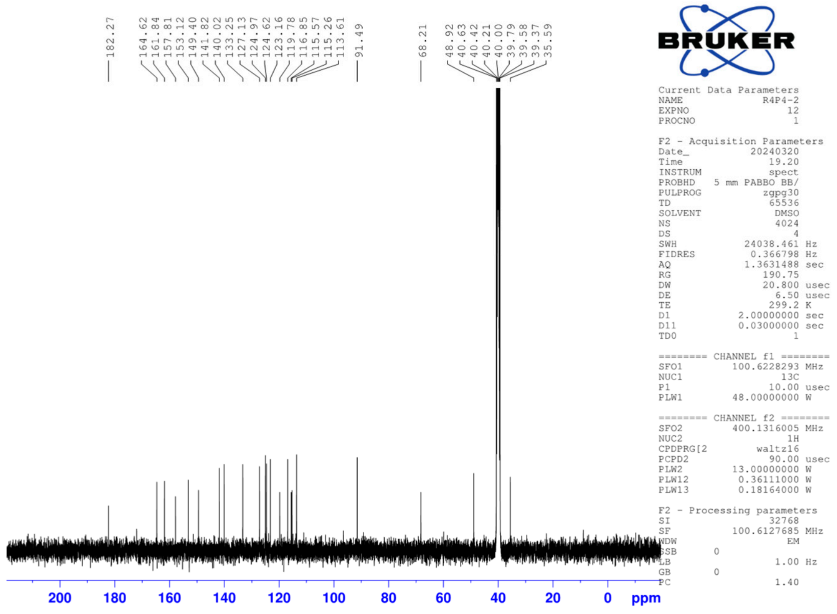

Figure S4. ^{1}H NMR spectrum (a) and ^{13}C NMR (b) of compound **6a4**

a)

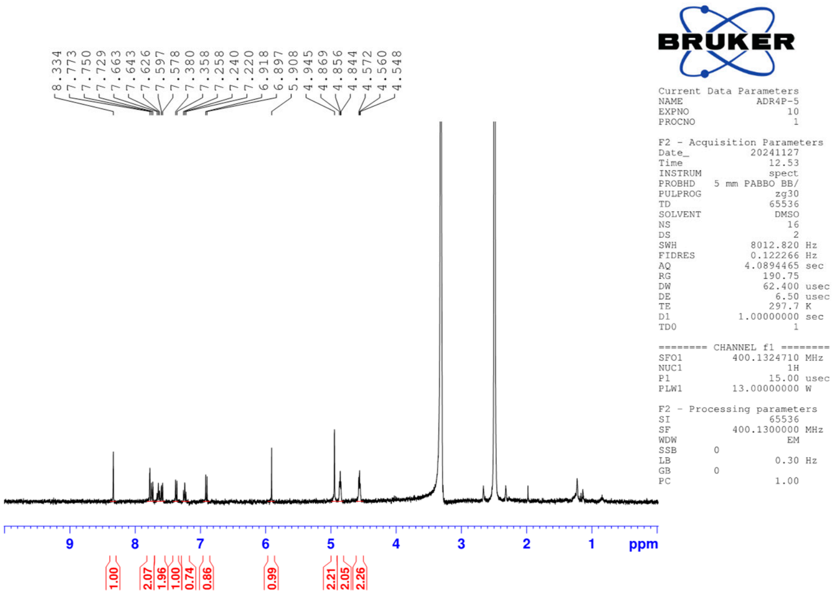

b)

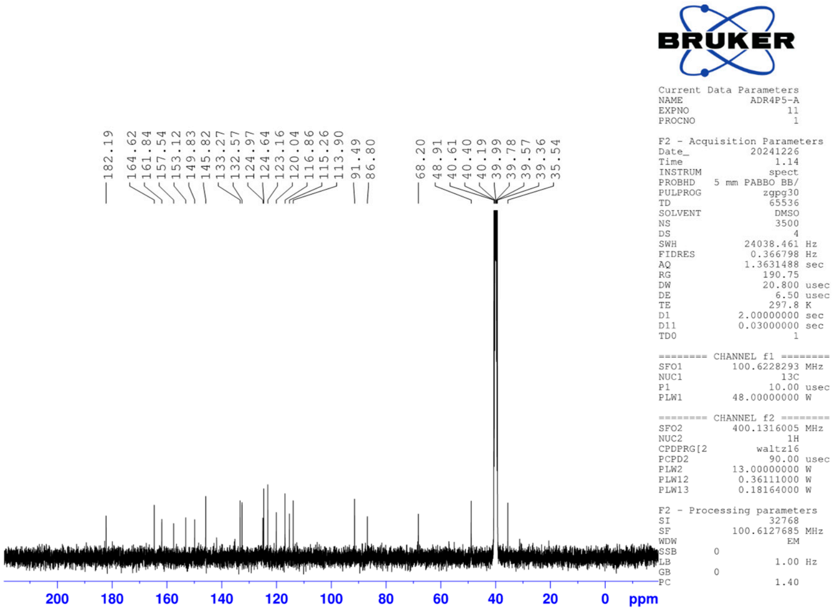

Figure S5. ^{1}H NMR spectrum (a) and ^{13}C NMR (b) of compound **6a5**

a)

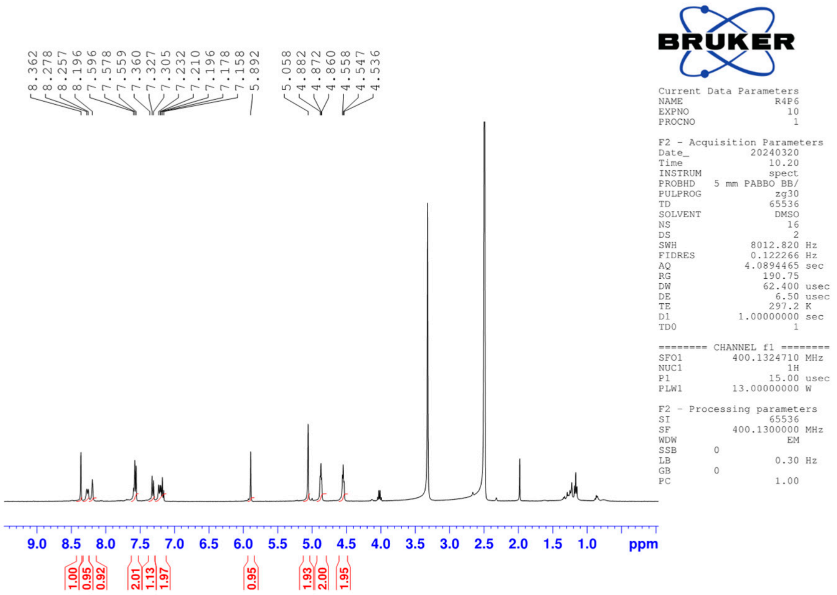

b)

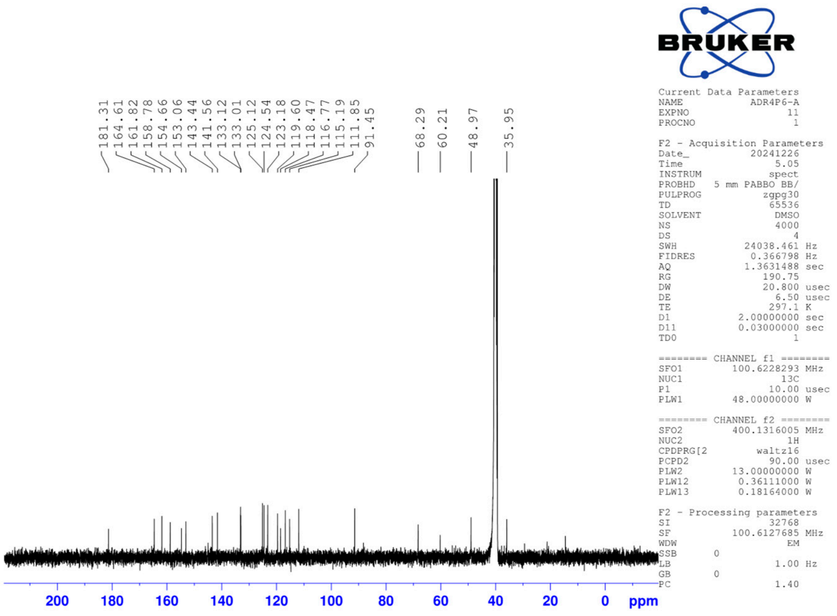

Figure S6. ^{1}H NMR spectrum (a) and ^{13}C NMR (b) of compound **6a6**

a)

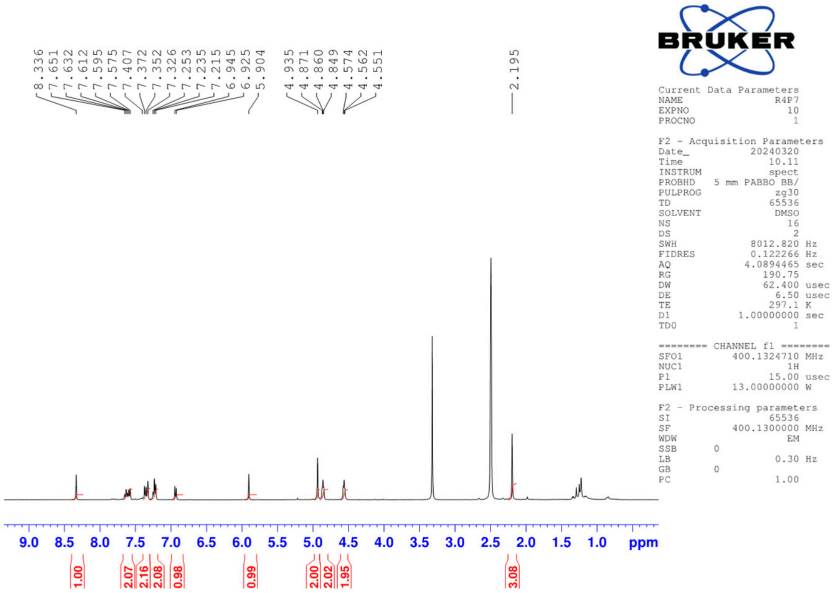

b)

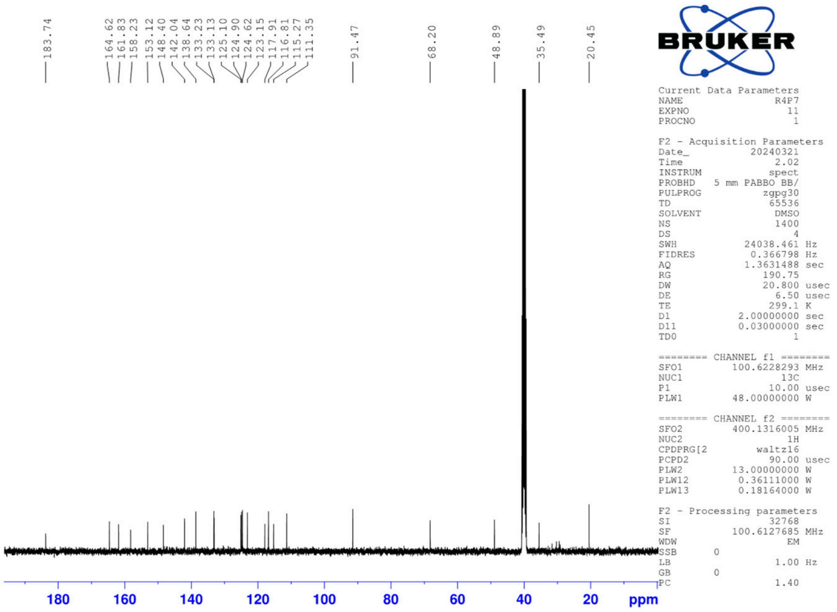

Figure S7. ^{1}H NMR spectrum (a) and ^{13}C NMR (b) of compound **6a7**

a)

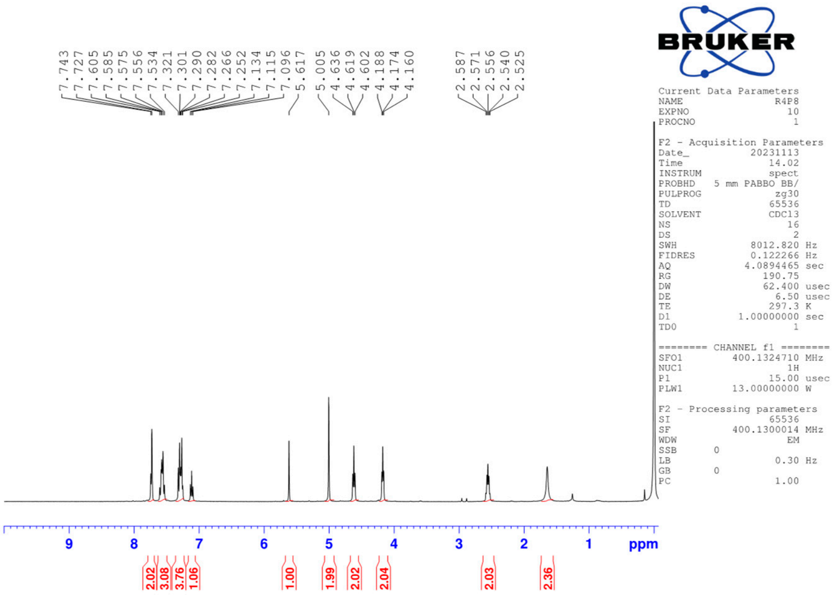

b)

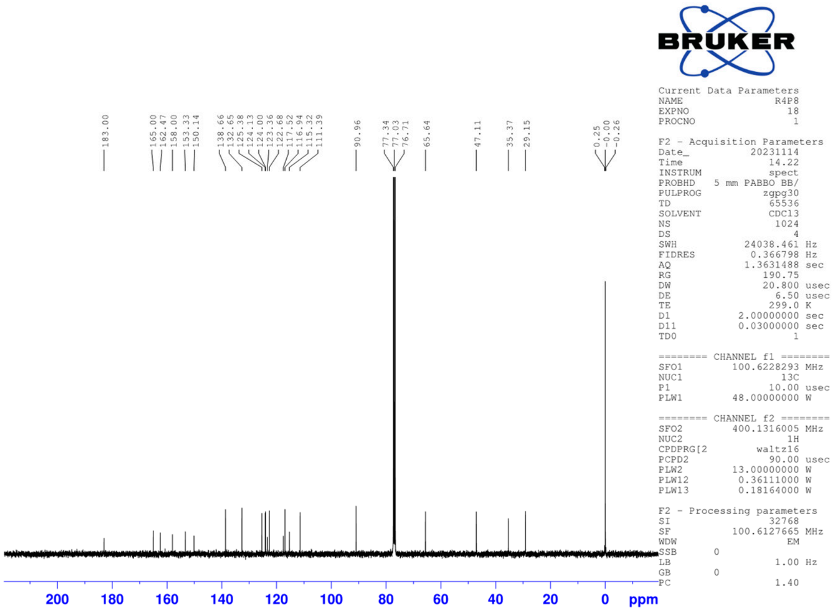

Figure S8. ^{1}H NMR spectrum (a) and ^{13}C NMR (b) of compound **6b1**

a)

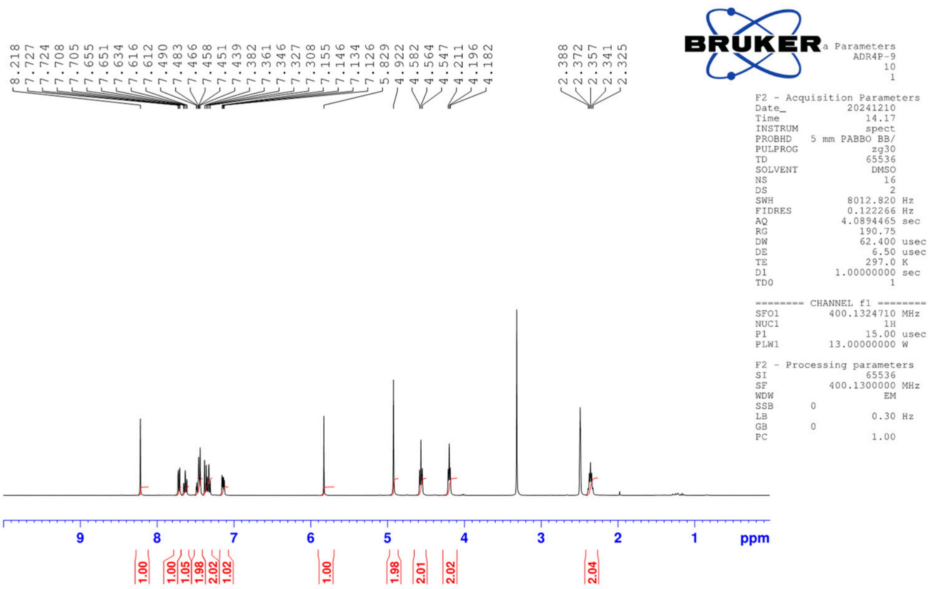

b)

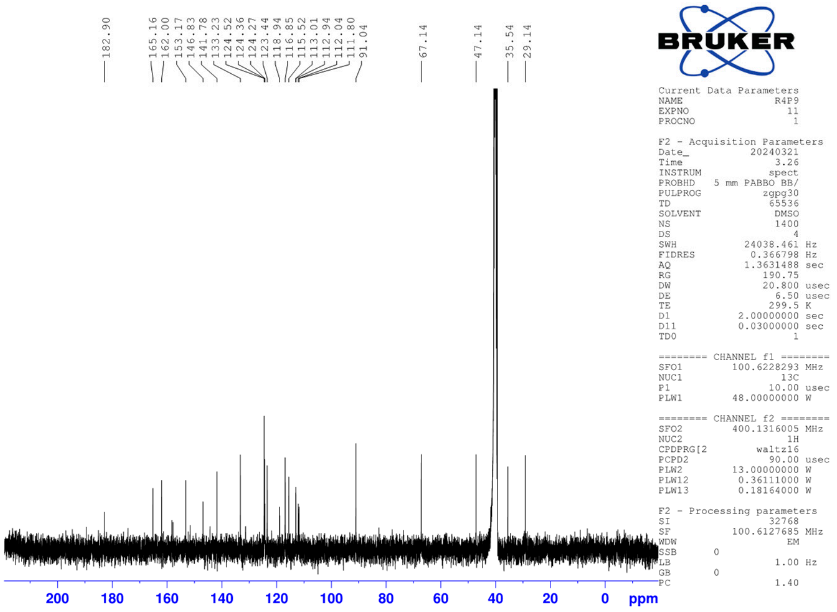

Figure S9. ^{1}H NMR spectrum (a) and ^{13}C NMR (b) of compound **6b2**

a)

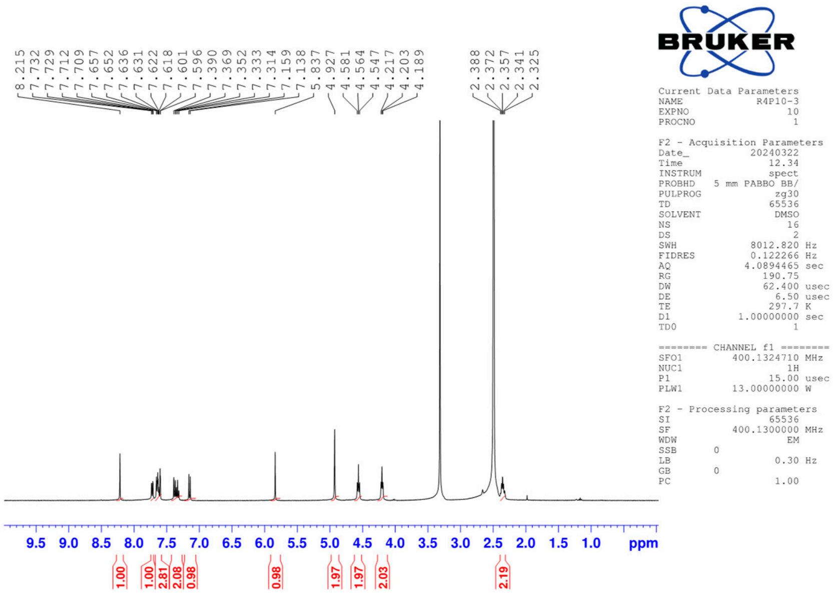

b)

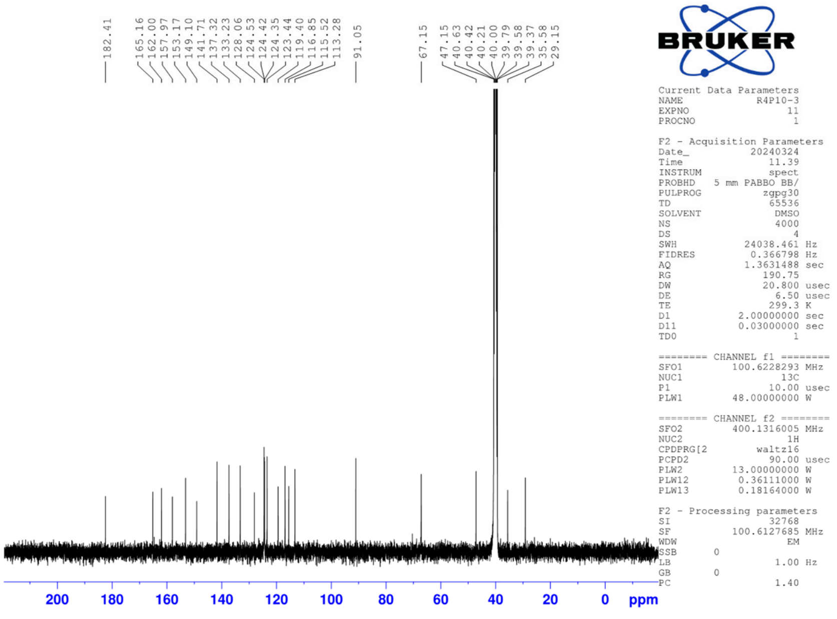

Figure S10. ^{1}H NMR spectrum (a) and ^{13}C NMR (b) of compound **6b3**

a)

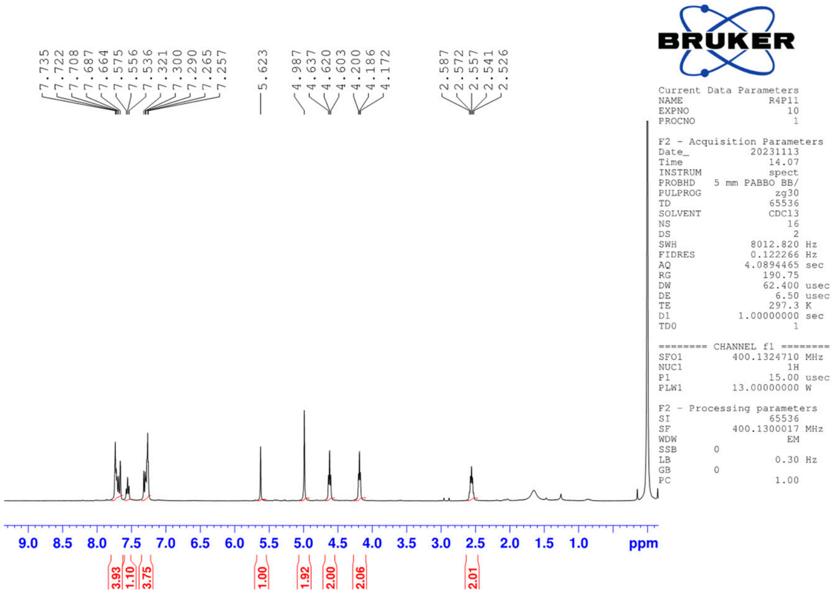

b)

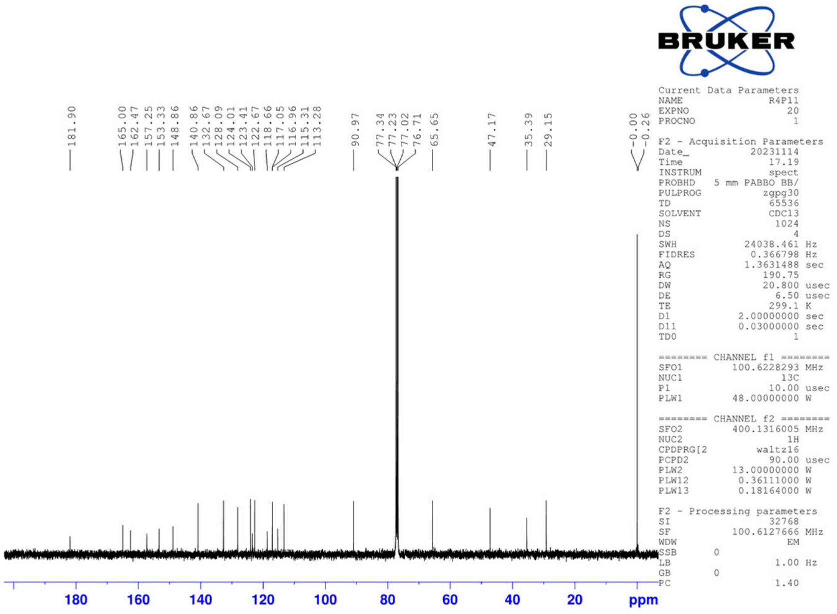

Figure S11. ^{1}H NMR spectrum (a) and ^{13}C NMR (b) of compound **6b4**

a)

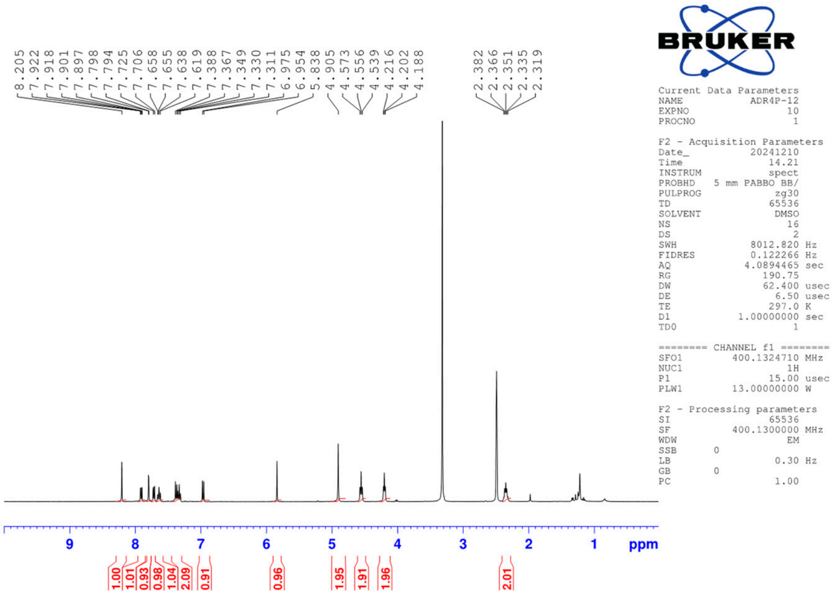

b)

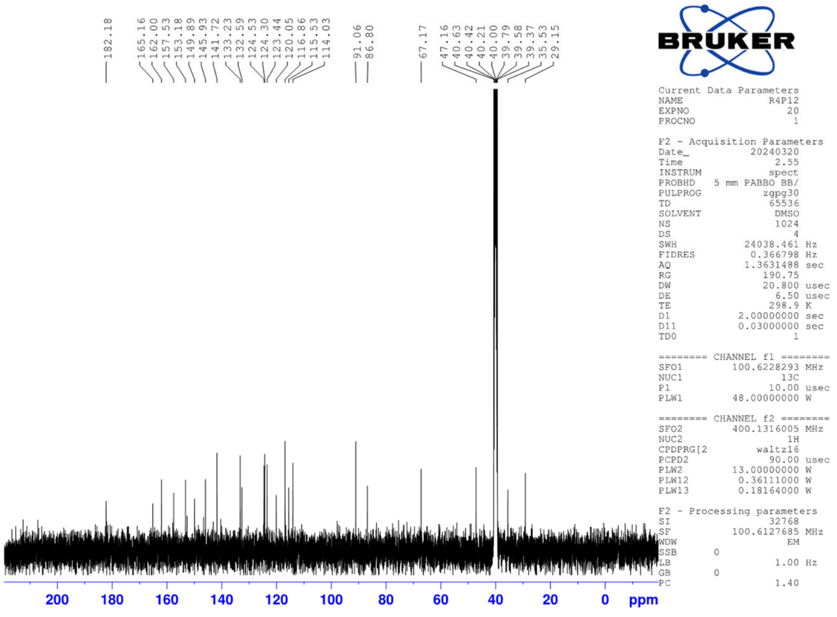

Figure S12. ^{1}H NMR spectrum (a) and ^{13}C NMR (b) of compound **6b5**

a)

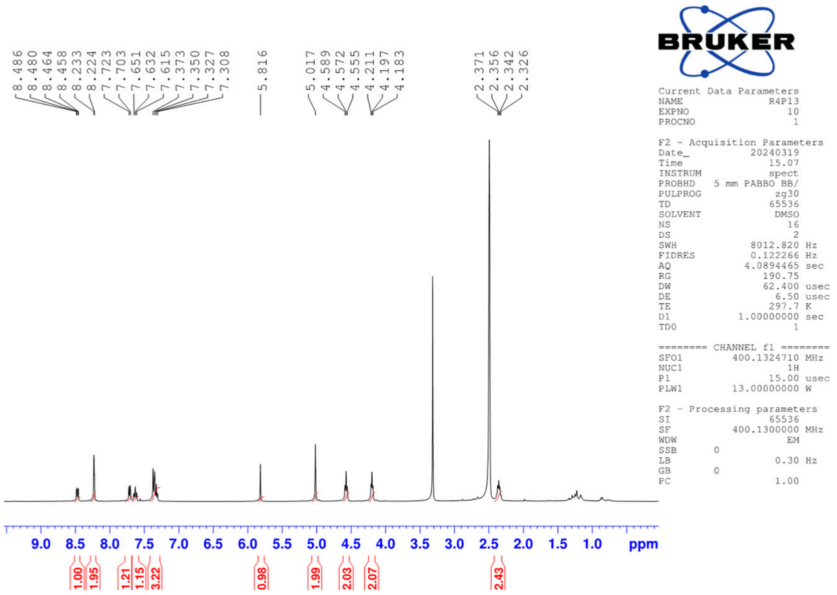

b)

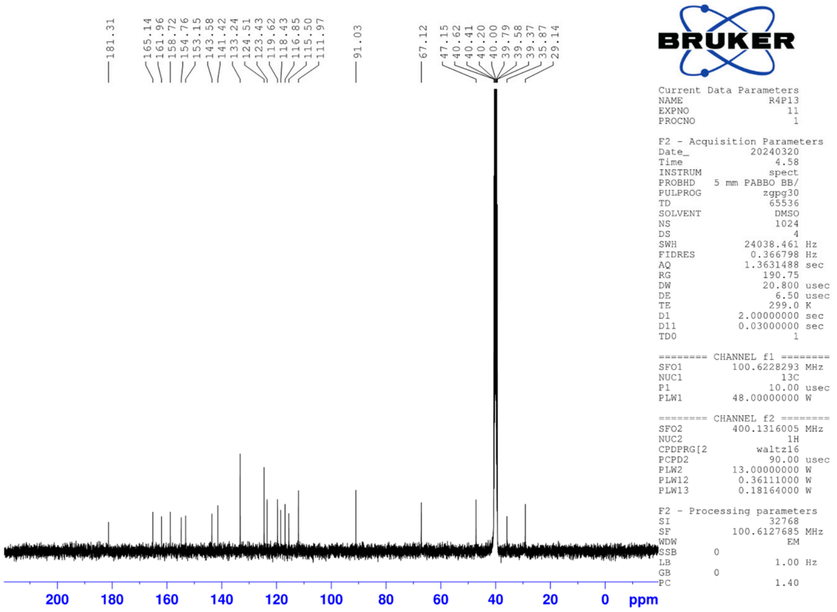

Figure S13. ^{1}H NMR spectrum (a) and ^{13}C NMR (b) of compound **6b6**

a)

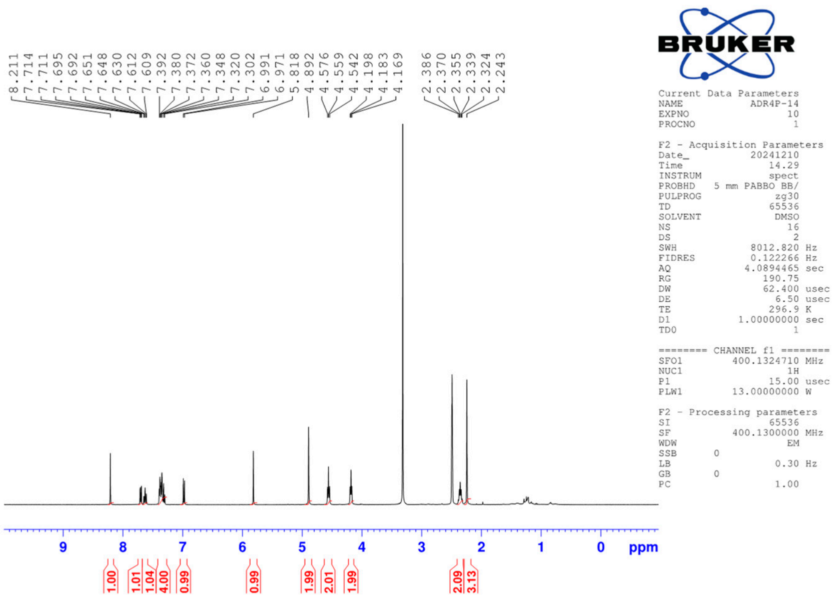

b)

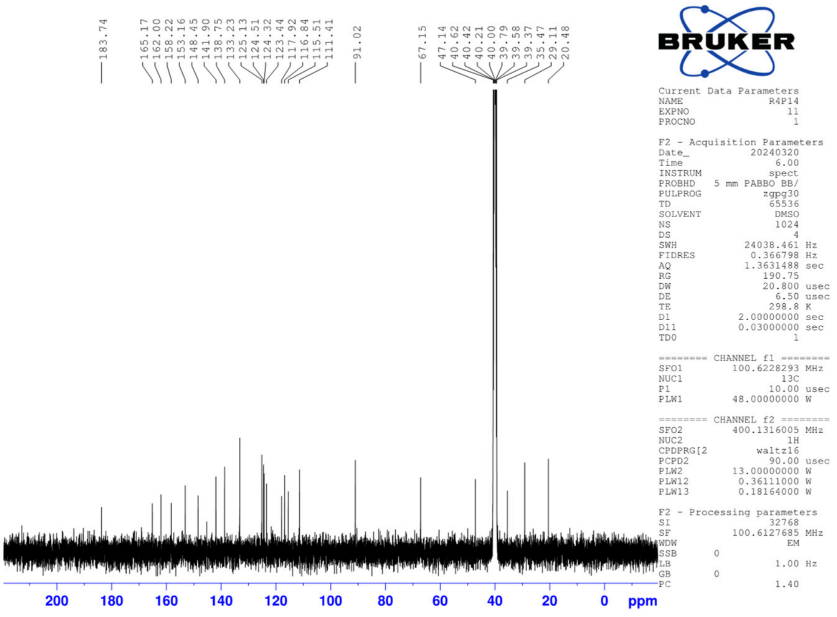

Figure S14. ^{1}H NMR spectrum (a) and ^{13}C NMR (b) of compound **6b7**

a)

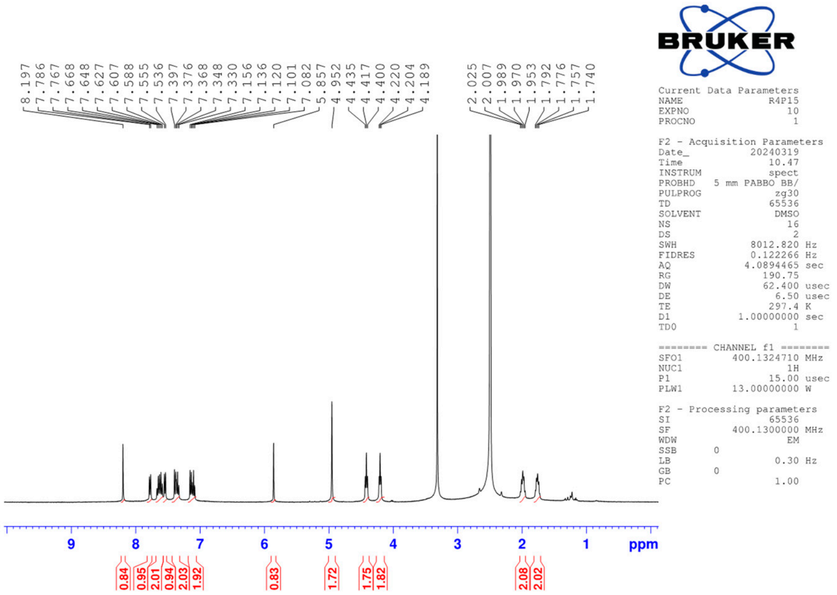

b)

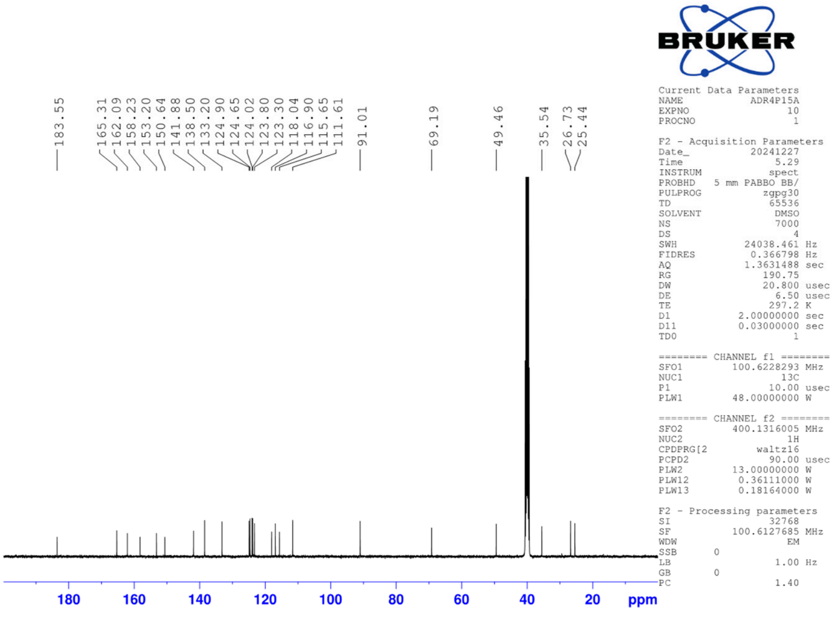

Figure S15. ^{1}H NMR spectrum (a) and ^{13}C NMR (b) of compound **6c1**

a)

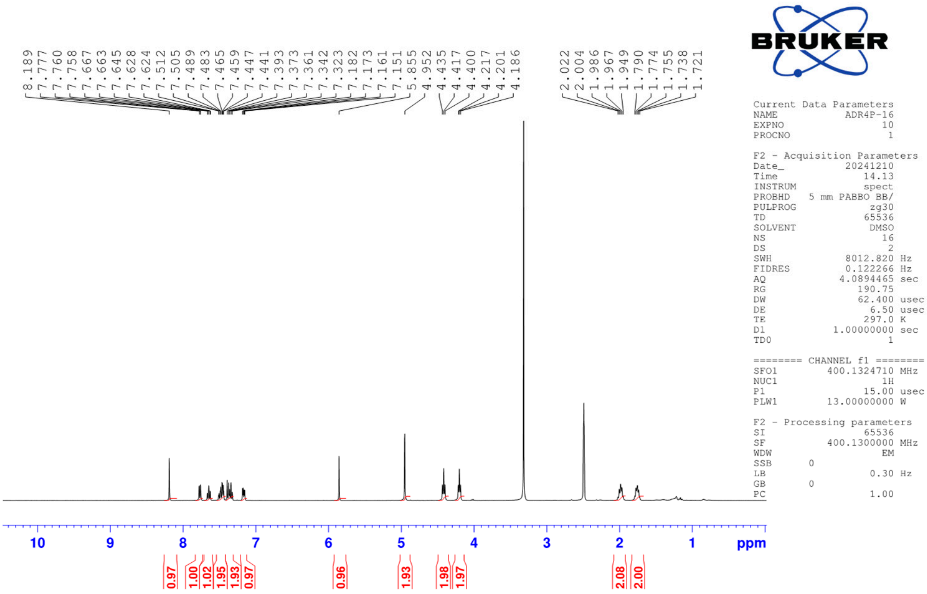

b)

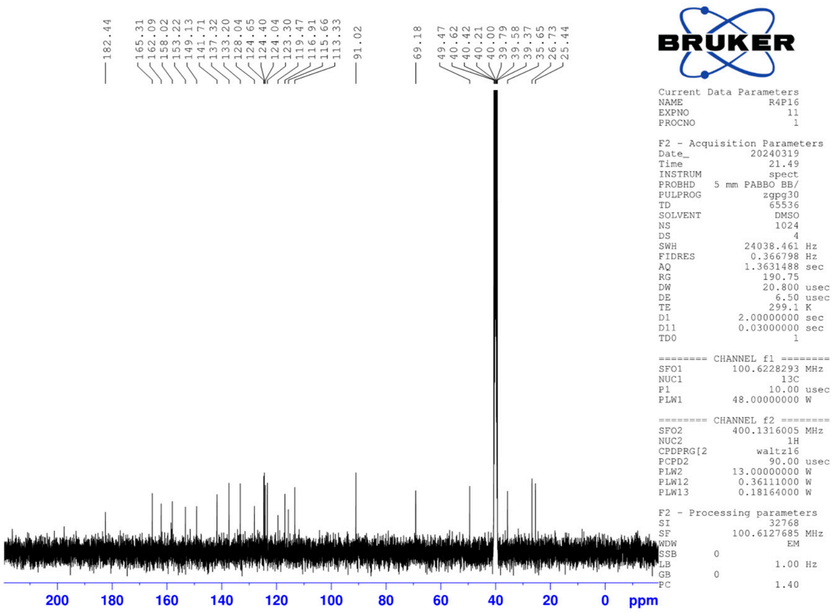

Figure S16. ^{1}H NMR spectrum (a) and ^{13}C NMR (b) of compound **6c2**

a)

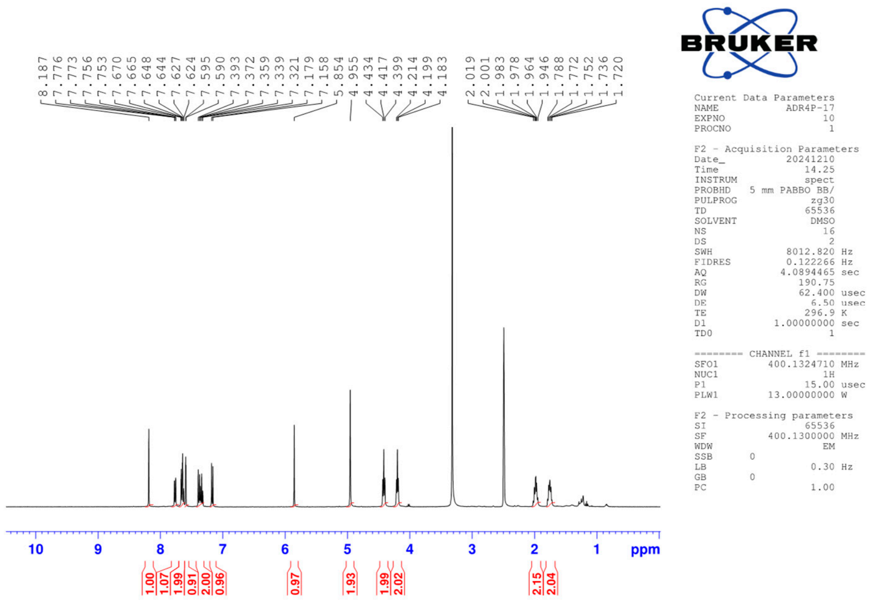

b)

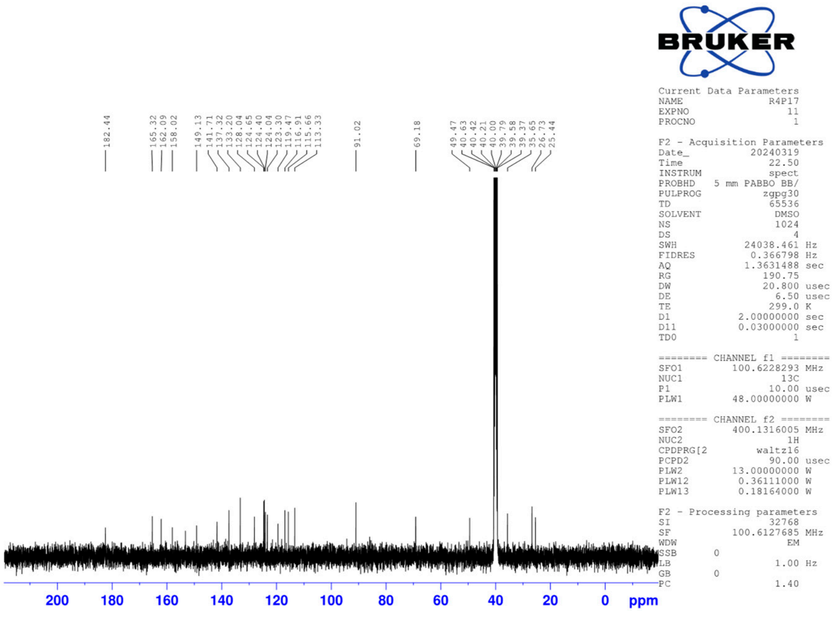

Figure S17. ^{1}H NMR spectrum (a) and ^{13}C NMR (b) of compound **6c3**

a)

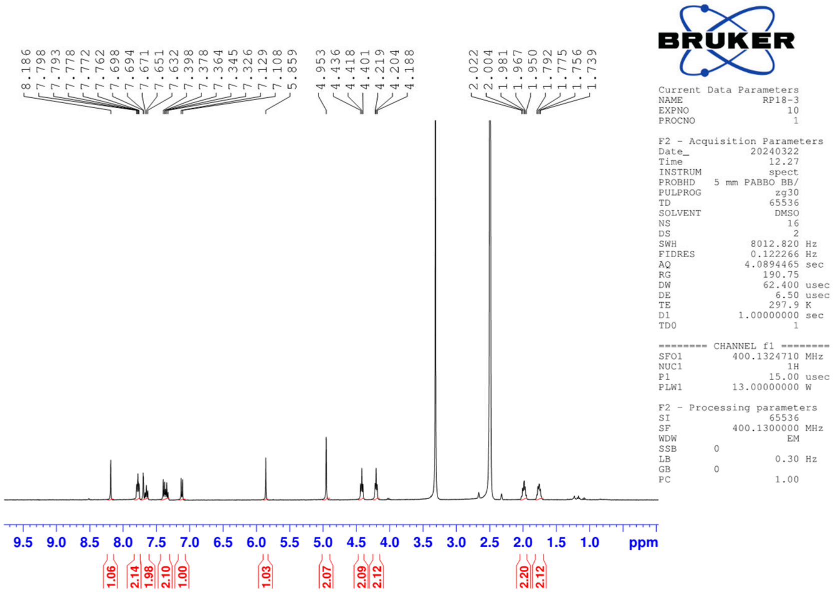

b)

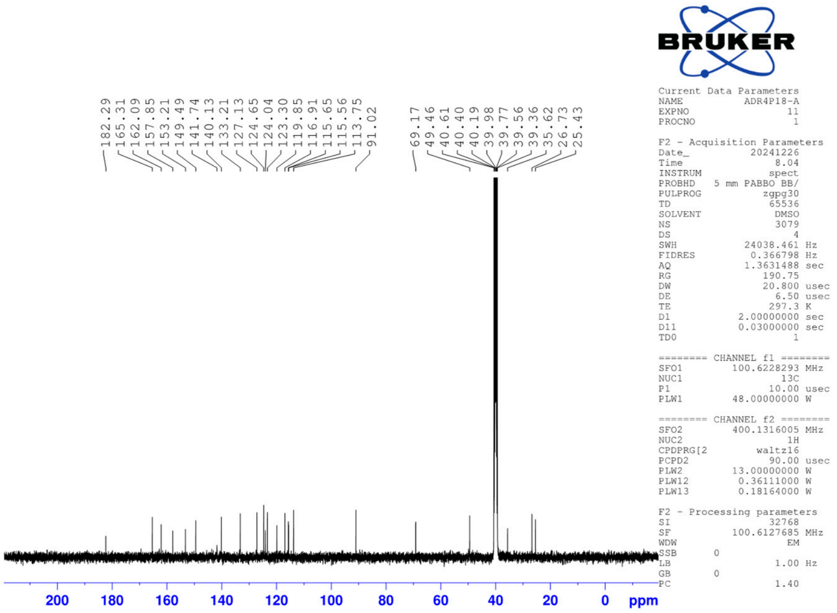

Figure S18. ^{1}H NMR spectrum (a) and ^{13}C NMR (b) of compound **6c4**

a)

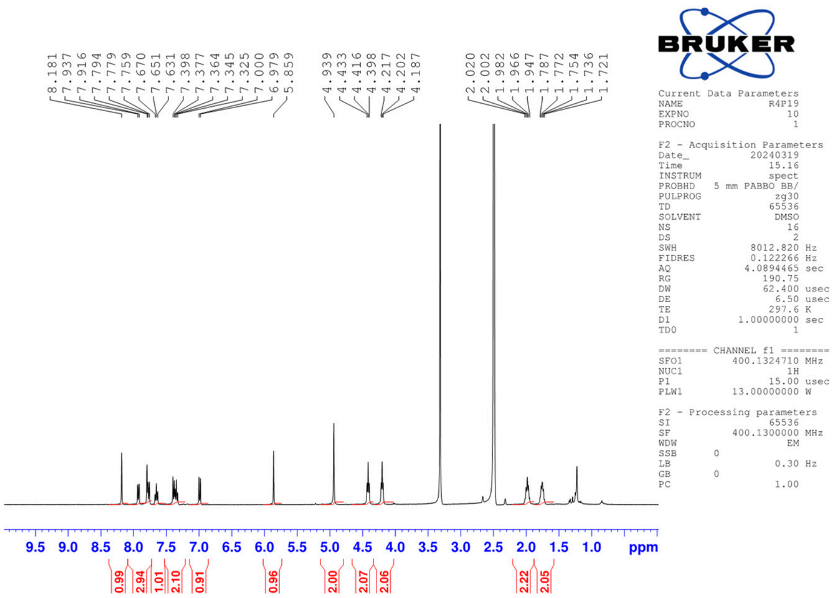

b)

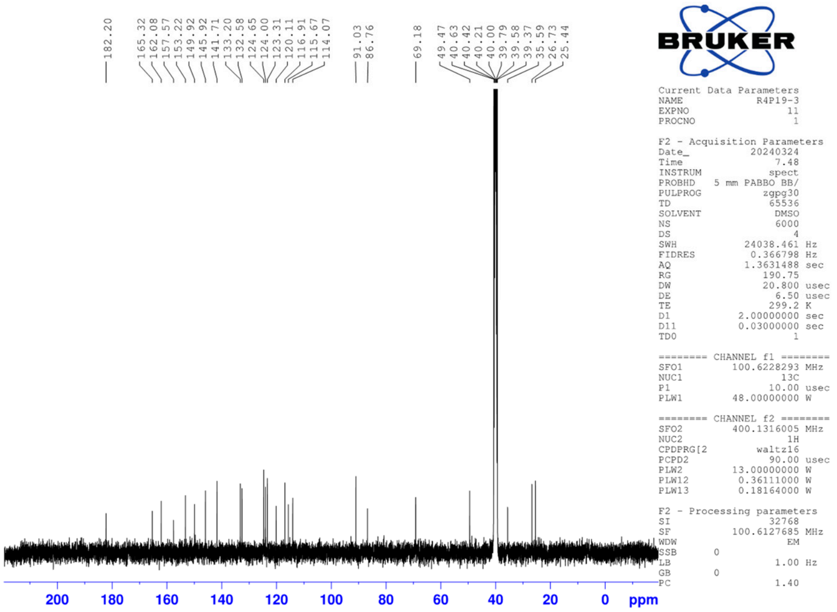

Figure S19. ^{1}H NMR spectrum (a) and ^{13}C NMR (b) of compound **6c5**

a)

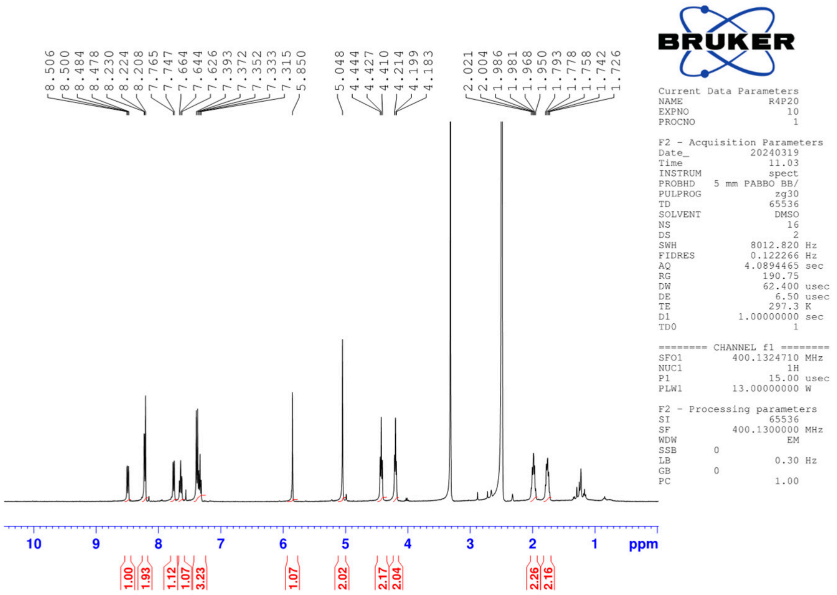

b)

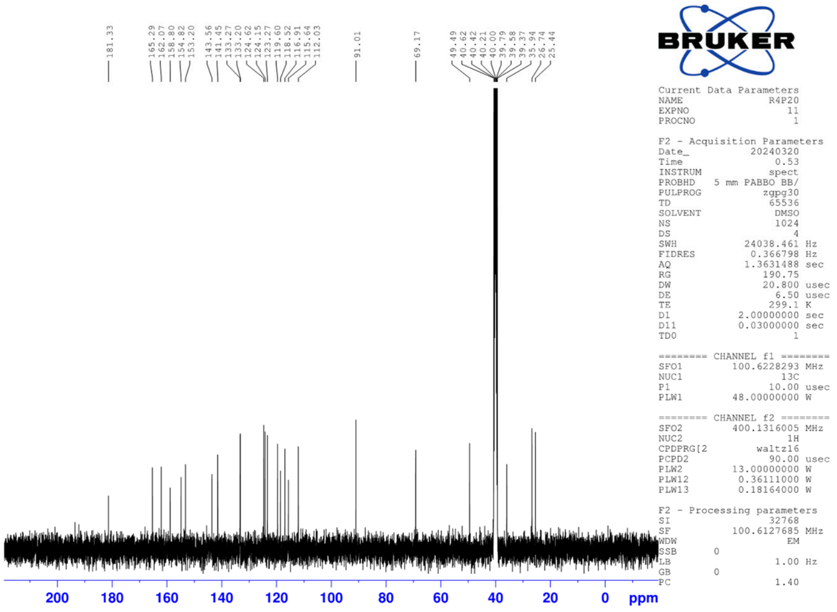

Figure S20. ^{1}H NMR spectrum (a) and ^{13}C NMR (b) of compound **6c6**

a)

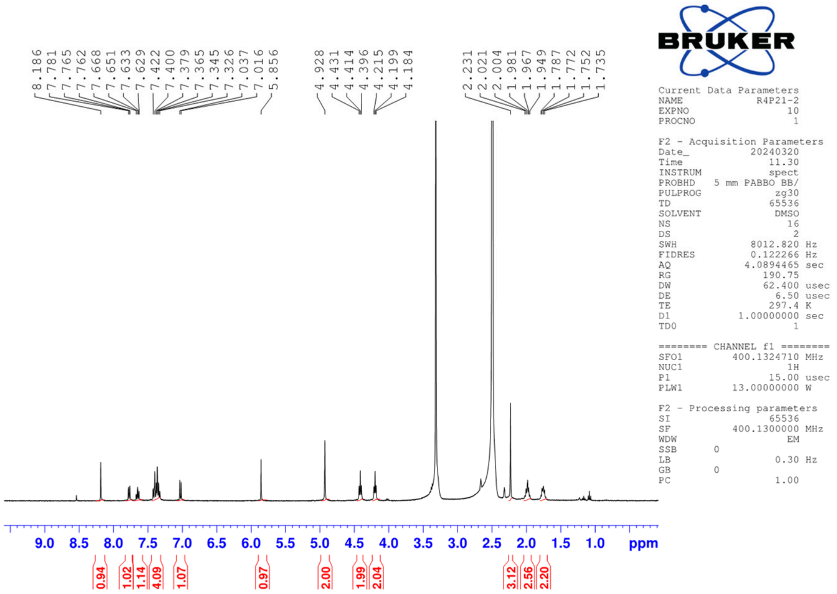

b)

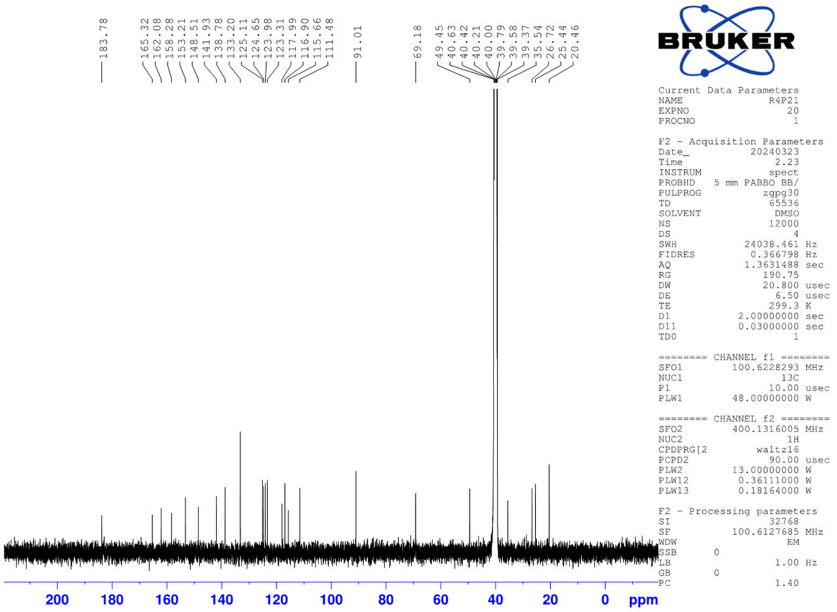

Figure S21. ^{1}H NMR spectrum (a) and ^{13}C NMR (b) of compound **6c7**

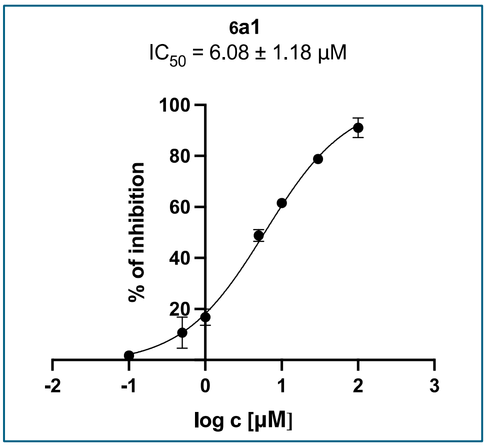

6a1
IC50 = 6.08 ± 1.18 µM
% of inhibition
log c [µM]

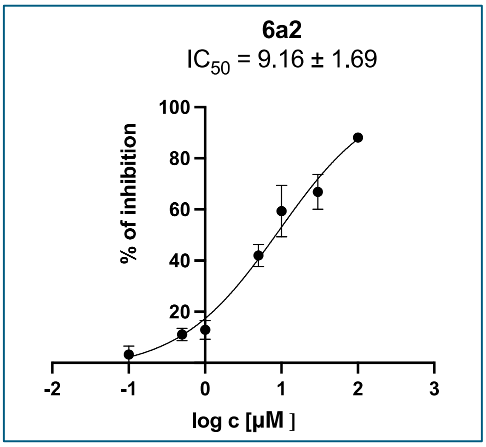

6a2
IC50 = 9.16 ± 1.69
% of inhibition
log c [µM]

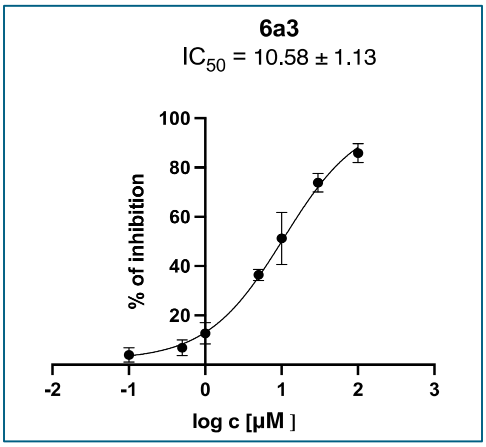

6a3
IC50 = 10.58 ± 1.13
% of inhibition
log c [µM]

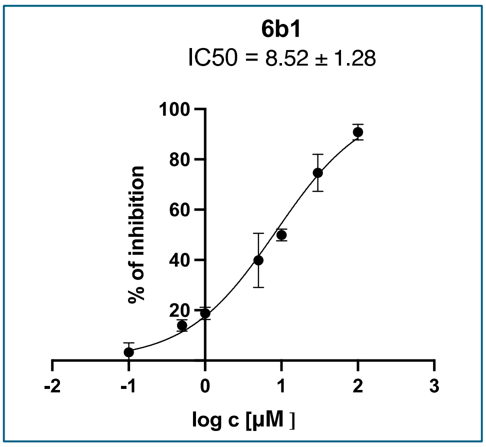

6b1
IC50 = 8.52 ± 1.28
% of inhibition
log c [µM]

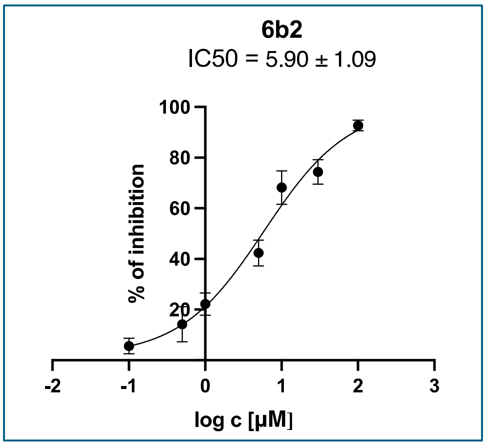

6b2
IC50 = 5.90 ± 1.09
% of inhibition
log c [µM]

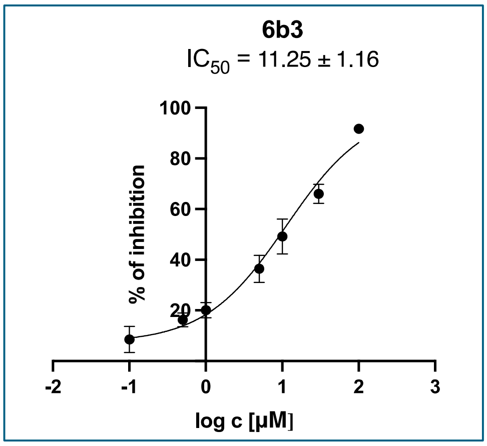

6b3
IC50 = 11.25 ± 1.16
% of inhibition
log c [µM]

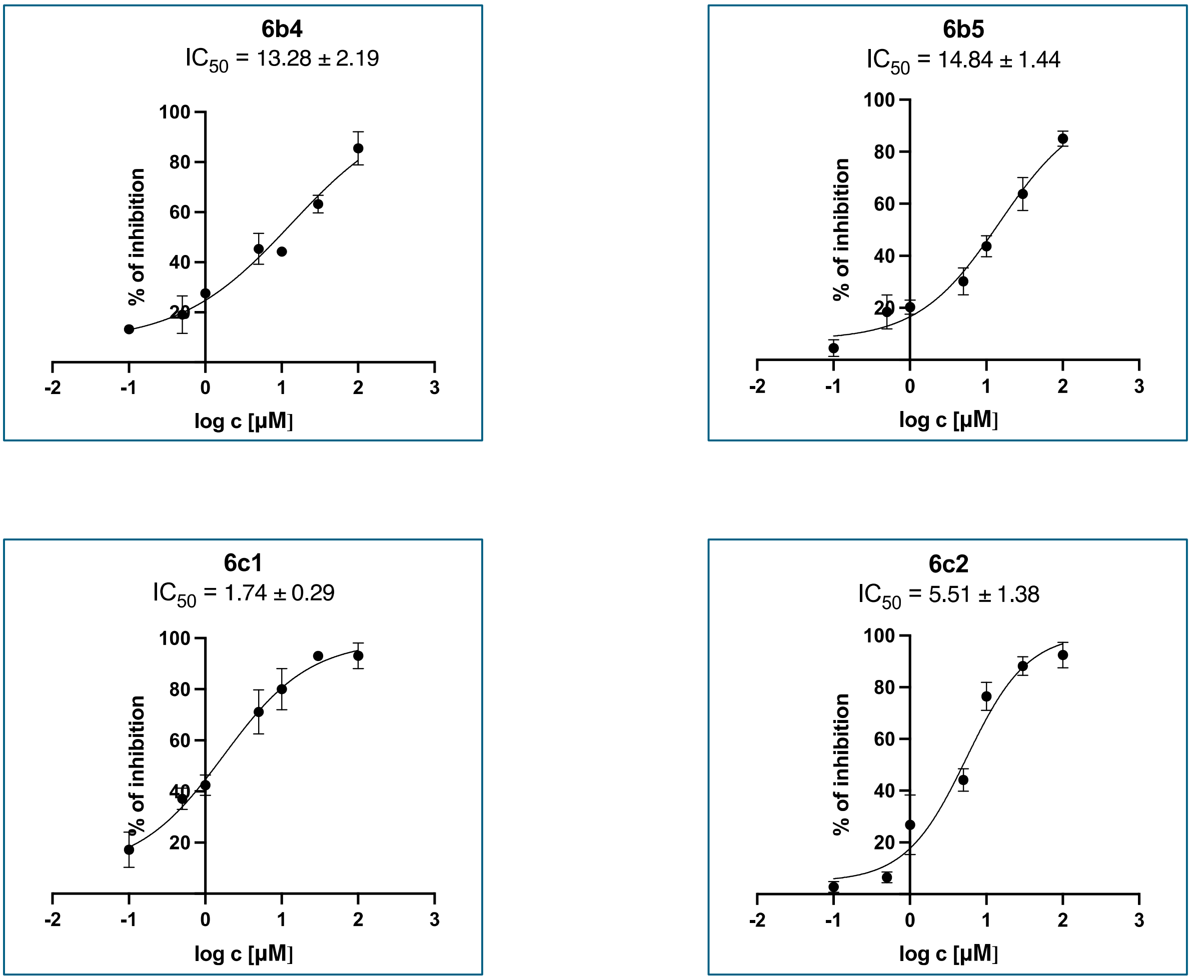

Figure S22. Dose–inhibition curves for the determination of IC_{50} values of compounds **6a1, 6a2, 6b1, 6b2, 6b3, 6b4, 6b5, 6c1,** and **6c2** against butyrylcholinesterase (BChE). Data points represent mean values of triplicate experiments, and curves were fitted using a four-parameter logistic model.

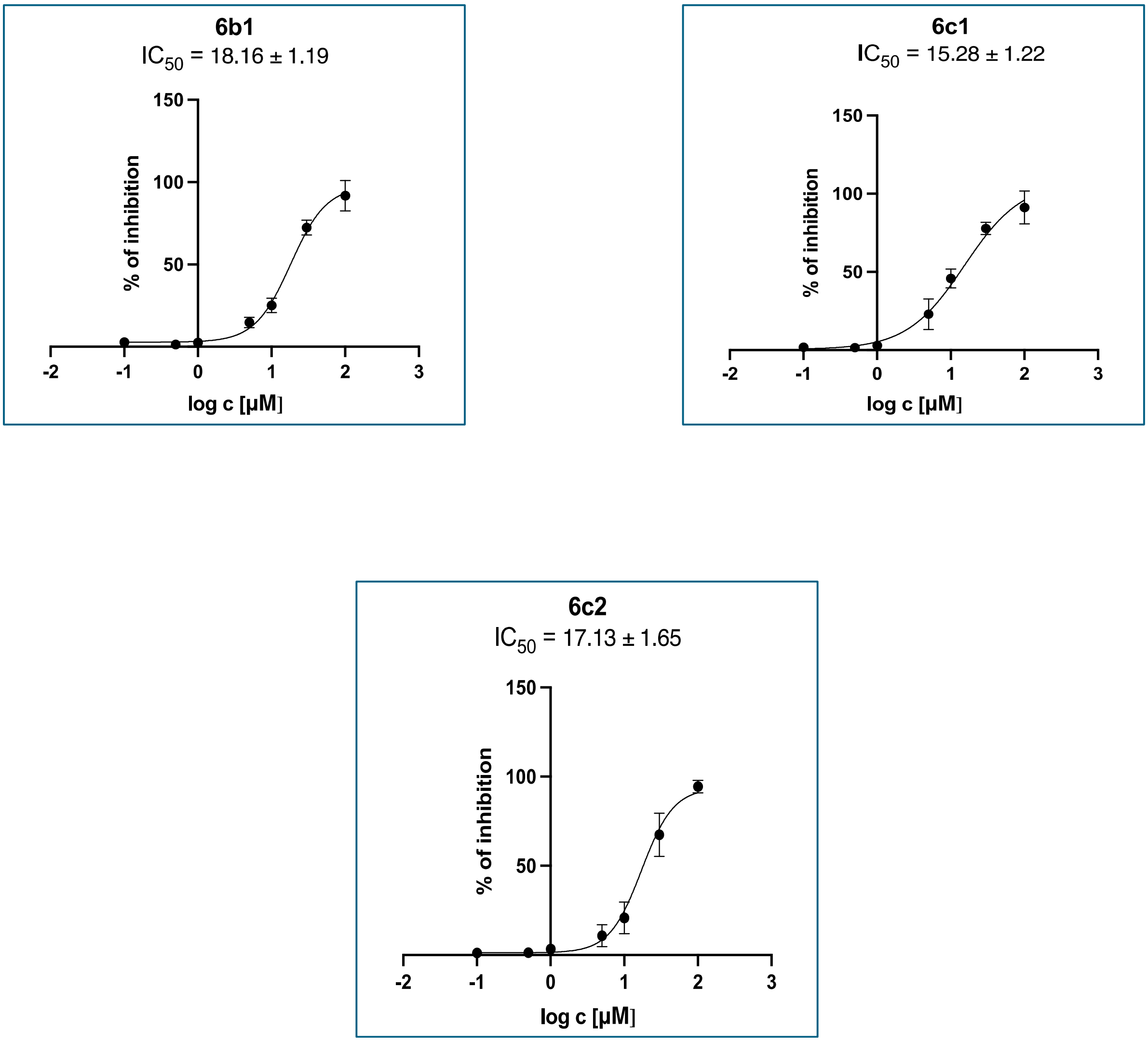

Figure S23. Dose–inhibition curves for the determination of IC_{50} values of compounds **6a1, 6c1,** and **6c2** against acetylcholinesterase (AChE). Data points represent mean values of triplicate experiments, and curves were fitted using a four-parameter logistic model.

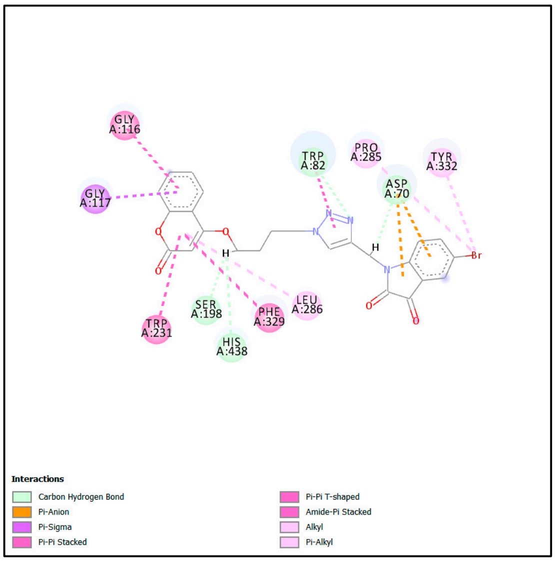

6c4

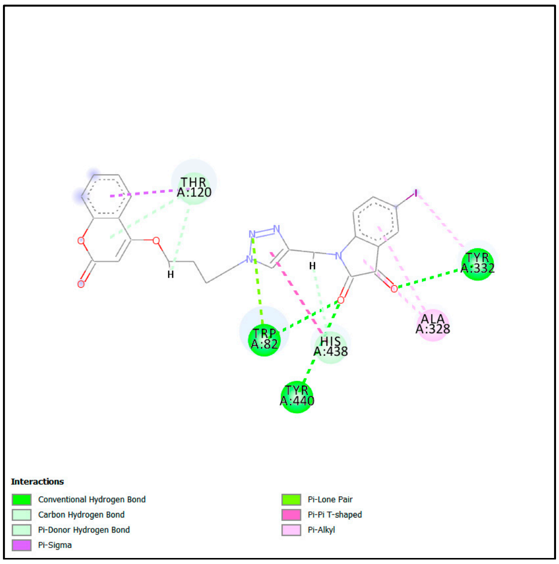

6c5

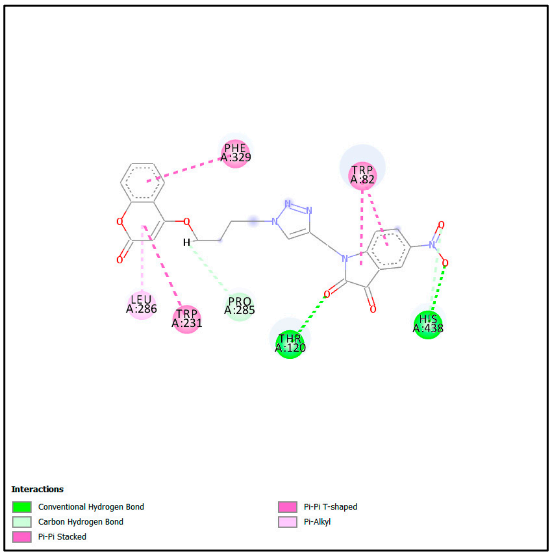

6c6

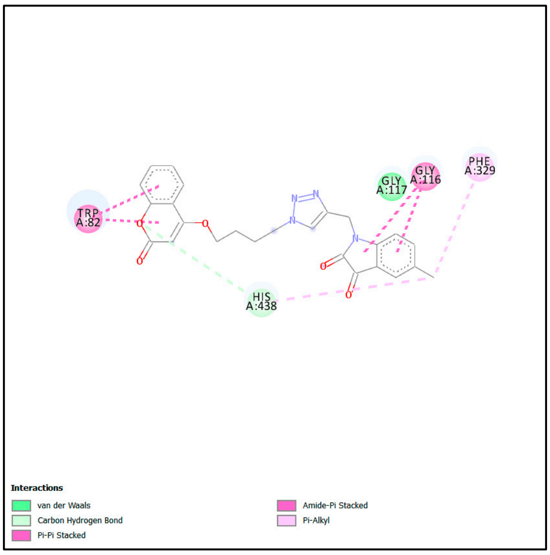

6c7

Figure S24. 2D interaction diagrams of representative inactive coumarin–triazole–isatin hybrids docked into the active site of human butyrylcholinesterase (PDB ID: 5K5E). Compounds **6c4, 6c5, 6c6** and **6c7** failed to establish key interactions with catalytic (Ser198, Trp82) and peripheral (Trp231, Phe329) residues, correlating with their low in vitro inhibitory activity.

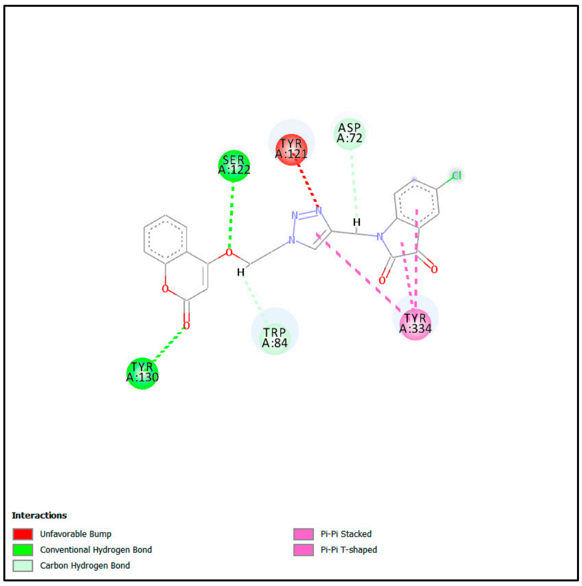

6a3

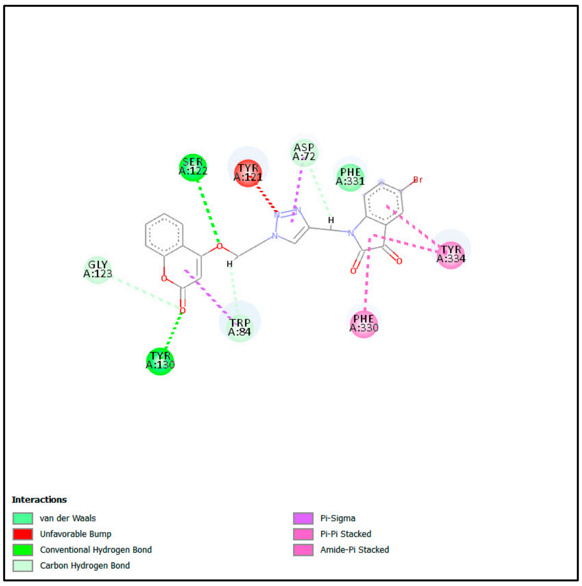

6a4

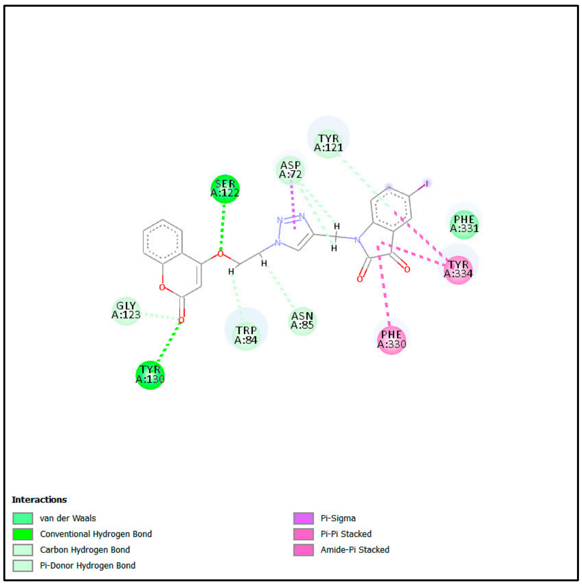

6a5

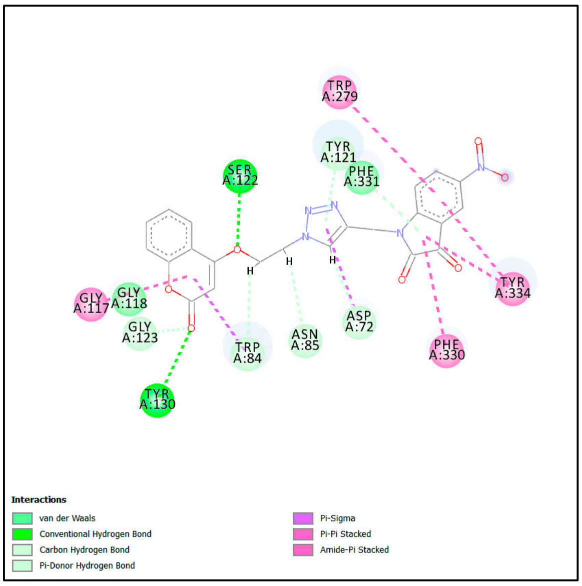

6a6

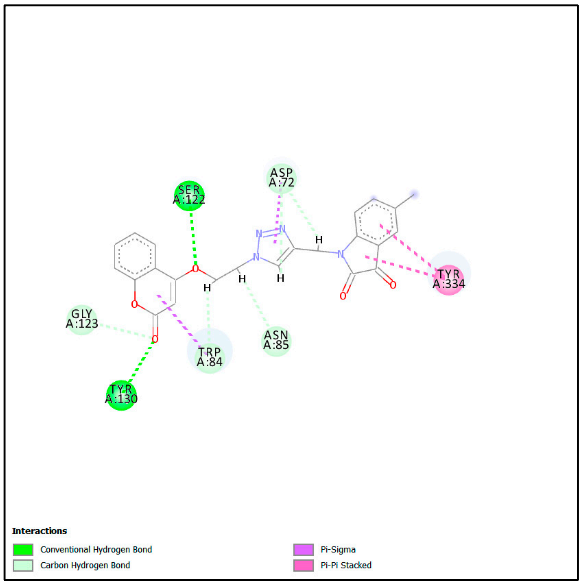

6a7

Figure S25. 2D interaction diagrams of representative inactive coumarin–triazole–isatin hybrids docked into the active site of *Torpedo californica* acetylcholinesterase (TcAChE, PDB ID: 5NAP). Compounds 6a3, 6a4, 6a5, 6a6 and 6a7 did not establish key interactions with crucial active-site residues (Trp84, Tyr121), consistent with their weak in vitro AChE inhibition.

Table S1. ChemGauss4 Scores of Compounds **6a1–6c7** and Donepezil Enantiomers Against hBChE (PDB ID: 5K5E) and TcAChE (PDB ID: 5NAP)

	**ChemGauss4 score**	
	**hBChE,** PDB ID: **5K5E**	***Tc*AChE**, PDB ID: **5NAP**
**6a1**	-17.09	-14.83
**6a2**	-17.40	-14.68
**6a3**	-17.62	-14.66
**6a4**	-17.72	-14.66
**6a5**	-18.42	-14.62
**6a6**	-17.74	-14.56
**6a7**	-17.33	-14.77
**6b1**	-16.54	-15.25
**6b2**	-16.06	-15.72
**6b3**	-16.56	-15.34
**6b4**	-16.96	-15.29
**6b5**	-17.24	-15.15
**6b6**	-16.77	-15.33
**6b7**	-16.76	-15.42
**6c1**	-15.44	-14.72
**6c2**	-15.22	-13.37
**6c3**	-15.36	-12.90
**6c4**	-14.93	-12.83
**6c5**	-14.81	-14.48
**6c6**	-13.97	-13.38
**6c7**	-15.02	-13.23
***S*-donepezil**	-17.26	-10.80
***R*-donepezil**	\	-10.74
